# Structure–activity relationships in a series of antiplasmodial thieno[2,3-*b*]pyridines

**DOI:** 10.1186/s12936-019-2725-y

**Published:** 2019-03-21

**Authors:** Andreas Masch, Abed Nasereddin, Arne Alder, Megan J. Bird, Sandra I. Schweda, Lutz Preu, Christian Doerig, Ron Dzikowski, Tim W. Gilberger, Conrad Kunick

**Affiliations:** 1 Institut für Medizinische und Pharmazeutische Chemie, Technische Universität Braunschweig, Beethovenstraße 55, 38106 Brunswick, Germany; 2 Department of Microbiology and Molecular Genetics, IMRIC, The Kuvin Center for the Study of Infectious and Tropical Diseases, The Hebrew University-Hadassah Medical School, 91120 Jerusalem, Israel; 3 Centre for Structural Systems Biology, Deutsches Elektronen-Synchrotron, Notkestraße 85, 22607 Hamburg, Germany; 4 Bernhard-Nocht Institute for Tropical Medicine, Bernhard-Nocht-Straße 74, 20359 Hamburg, Germany; 5 Biomedicine Discovery Institute, Infection & Immunity Program, Department of Microbiology, Monash University, Clayton, VIC 3800 Australia; 6 Center of Pharmaceutical Engineering (PVZ), Technische Universität Braunschweig, Franz-Liszt-Straße 35A, 38106 Brunswick, Germany; 7 Present Address: Genomics Applications Laboratory, Core Research Facility, Faculty of Medicine, The Hebrew University-Hadassah Medical School, 91120 Jerusalem, Israel; 8 Present Address: Centre for Chronic, Inflammatory and Infectious Diseases, School of Health and Biomedical Sciences, RMIT University, Bundoora, VIC 3083 Australia

**Keywords:** Anti-malaria drugs, Atropisomers, Axial chirality, Drug discovery, Malaria, *Pf*GSK-3, *Plasmodium falciparum*, Protein kinase, Thienopyridines, Thorpe cyclization

## Abstract

**Background:**

Malaria is one of the most prevalent tropical infectious diseases. Since recently cases of artemisinin resistance were reported, novel anti-malarial drugs are required which differ from artemisinins in structure and biological target. The plasmodial glycogen synthase kinase-3 (*Pf*GSK-3) was suggested as a new anti-malarial drug target. 4-Phenylthieno[2,3-*b*]pyridines were previously identified as selective *Pf*GSK-3 inhibitors with antiplasmodial activity. The present study aims at identifying a molecular position on this scaffold for the attachment of side chains in order to improve solubility and antiplasmodial activity. Furthermore, the role of axial chirality in the compound class for antiplasmodial activity and *Pf*GSK-3 inhibition was investigated.

**Methods:**

4-Phenylthieno[2,3-*b*]pyridines with substituents in 4-position of the phenyl ring were docked into the ATP binding site of *Pf*GSK-3. The compounds were synthesized employing a Thorpe reaction as final step. The enantiomers of one congener were separated by chiral HPLC. All derivatives were tested for inhibition of asexual erythrocytic stages of transgenic NF54-*luc Plasmodium falciparum.* Selected compounds with promising antiplasmodial activity were further evaluated for inhibition of HEK293 cells as well as inhibition of isolated *Pf*GSK-3 and *Hs*GSK-3. The kinetic aqueous solubility was assessed by laser nephelometry.

**Results:**

The para position at the 4-phenyl ring of the title compounds was identified as a suitable point for the attachment of side chains. While alkoxy substituents in this position led to decreased antiplasmodial activity, alkylamino groups retained antiparasitic potency. The most promising of these congeners (**4h**) was investigated in detail. This compound is a selective *Pf*GSK-3 inhibitor (versus the human GSK-3 orthologue), and exhibits improved antiplasmodial activity in vitro as well as better solubility in aqueous media than its unsubstituted parent structure. The derivative **4b** was separated into the atropisomers, and it was shown that the (+)-enantiomer acts as eutomer.

**Conclusions:**

The attachment of alkylamino side chains leads to the improvement of antiplasmodial activity and aqueous solubility of selective *Pf*GSK-inhibitors belonging to the class of 4-phenylthieno[2,3-*b*]pyridines. These molecules show axial chirality, a feature of high impact for biological activity. The findings can be exploited for the development of improved selective *Pf*GSK-3 inhibitors.

**Electronic supplementary material:**

The online version of this article (10.1186/s12936-019-2725-y) contains supplementary material, which is available to authorized users.

## Background

In spite of successful efforts to fight malaria in recent years, the infection remains one of the most widespread and dangerous tropical diseases. As a result of improved diagnosis, intensified vector control and infection prophylaxis, the prevalence of malaria significantly declined between the years 2000 and 2015 in terms of both morbidity and mortality. This positive development recently came to a standstill in 2016. 216 million cases were reported globally, an increase of five million cases over the count in the previous year. Furthermore, the rate of fatalities resulting from the infection is no longer decreasing, but stagnating at approximately 440,000 per year [1]. The most lethal form of the disease is caused by the parasite *Plasmodium falciparum.* Artemisinin-based combination therapy (ACT) is recommended for the treatment of *P. falciparum* malaria [1]. Unfortunately, resistant *P. falciparum* strains have been reported against all deployed anti-malarial drugs, including artemisinins. Artemisinin resistance was initially observed in South-East Asia [2] and is currently still restricted to this geographic area [3]. However, spread of artemisinin resistance to other parts of the world, especially sub-Saharan Africa (where most of the *P. falciparum* infections occur), would create a desperate situation [4]. Novel drugs for prophylaxis and treatment of malaria are urgently required, either as replacement or additional partner for artemisinin-based combinations. To prevent cross-resistance, new medicines should have an untapped mode of action, and therefore be based on chemotypes distinct from artemisinins or other established anti-malarial drugs [5].

**Fig. 1:**
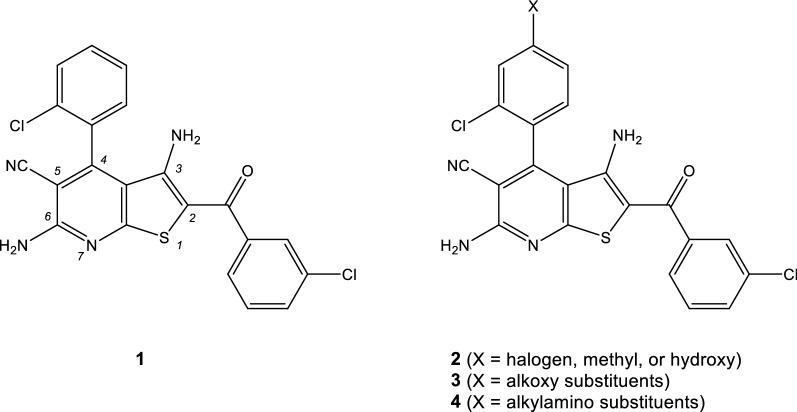
Left: Structures of the antiplasmodial selective *Pf*GSK-3 inhibitor **1** [25] with indicated locants at the heterocyclic parent scaffold (right) and derivatives **2**–**4** with sidechains attached to the para position of the 4-phenyl ring as listed in Table 1

Plasmodial kinases have consistently been suggested as biological targets for antimalarial drugs [6–13], and a number of medicinal chemistry campaigns have been performed to develop kinase inhibitors as antiplasmodial compounds [14]. MMV390048 was identified from a phenotypic screening campaign. The compound inhibits the *Plasmodium* phosphatidylinositol 4-kinase (*Pf*PI4K) [15] and is currently in clinical trials as an anti-malaria drug [14]. The kinome of *P. falciparum* includes 65 kinases related to the eukaryotic protein kinase family, of which 36 were found to be essential for the erythrocytic schizogony. Among these essential kinases is the plasmodial glycogen synthase kinase-3 (*Pf*GSK-3), the orthologue of the human serine/threonine kinase GSK-3 [16]. An important link in the signaling pathway downstream of the insulin receptor, *Hs*GSK-3 deactivates glycogen synthase (GS) by phosphorylation. The kinase is also an intracellular part of the Wnt pathway which regulates cell fate during embryonic development [17]. Because several human diseases are connected to hyperactivity of GSK-3 (e.g. Alzheimer’s disease, diabetes and cancer), a variety of GSK-3 inhibitors were developed as potential drugs [18]. The manifold roles of GSK-3 in mammalian organisms led to speculations about the function of *Pf*GSK-3 in the parasite, e.g. regulation of the cell cycle, cellular differentiation, and metabolism [19, 20]. The plasmodial gene homologue *Pf*GSK-3 of mammalian GSK-3 was identified and cloned by PCR [19]. The sequence characterization of *Pf*GSK-3 and the comparison with the human orthologue revealed a considerable degree of similarity within the catalytic domain. Important amino acids of the active site of *Hs*GSK-3 (Lys85, Glu97, Asp181 and Asp200) are conserved in *Pf*GSK-3 (as Lys108, Glu20, Asp206 and Asp225, respectively). A main difference between the two proteins is the lack of a phosphorylation site in *Pf*GSK-3 analogous to Ser9 in *Hs*GSK-3β. Once phosphorylated, the latter residue is responsible for *Hs*GSK-3 auto-inhibition through binding to the P + 4 phosphate binding cleft present in the enzyme [21]. Furthermore, *Pf*GSK-3 has an *N*-terminal extension which is not present in mammalian GSK-3 [19]. Recently it has been shown that phosphorylation of the apical membrane antigen 1 (AMA1) of *P. falciparum* by *Pf*GSK-3 is required for efficient invasion of red blood cells by merozoites [22]. Since *Pf*GSK-3 appears to be essential, GSK-3 inhibitors were suggested as antiplasmodial agents [19]. An initial study with a set of established GSK-3 inhibitors revealed that some of these chemotypes (e.g. hymenialdisine and indirubin-3′-monoxime) do indeed affect *Pf*GSK-3 activity, but do not distinguish between the plasmodial and the mammalian orthologue [19]. However, representatives of the paullone class of compounds [23, 24] exhibited a remarkable preference for inhibition of mammalian GSK-3, thus indicating that species selectivity may be achievable [19]. Based on this assumption, a high throughput screening was carried out by testing compound libraries against both *Pf*GSK-3, using the mammalian orthologue in a counter screen [25]. This produced a small number of hit compounds characterized by a thieno[2,3-*b*]pyridine parent scaffold, which displayed selectivity against the plasmodial (versus human) enzyme. Chemical modification of this scaffold identified compound **1** (Fig. 1), which selectively inhibited *Pf*GSK-3 in an ATP-competitive manner and showed antiparasitic activity on *P. falciparum* erythrocytic stages in vitro [25]. Further structure modification of **1** revealed rather narrow structure–activity relationships. For example, the ortho-halogen substituent on the 4-phenyl ring was required for *Pf*GSK-3 inhibition, replacement of the carbonyl linker by an amide function and annulation at positions 5,6 with cyclohexane led to decreased kinase inhibitory activity, and exchange of the benzoyl element for a nitrile group decreased selectivity [25].

**Fig. 2:**
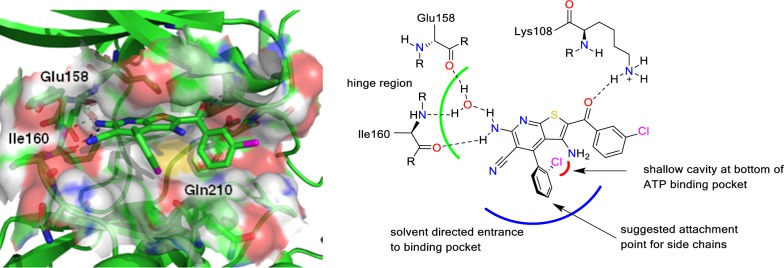
Orientation of thieno[2,3-*b*]pyridine **1** accommodated in the ATP-binding pocket of a *Pf*GSK-3 homology model as predicted by docking experiments. Left: 3D-illustration (generated with PyMol, vers. V0.99 [29]). Right: 2D-sketch. Dashed black lines indicate hydrogen bonds. Colour code: blue, nitrogen; red, oxygen; yellow, sulfur; magenta, chlorine

An unfavourable feature of compound **1** and similar molecules is the poor solubility in aqueous media, a property of high relevance for the development of drugs intended for oral application. The poor solubility of **1** is probably caused only by high lipophilicity but may also be a consequence of a high insaturation count. Since not a single carbon atom in **1** is sp^3^ hybridized, the fraction sp^3^ (Fsp^3^ = number of sp^3^ hybridized carbons/total carbon count) is zero, a very unfavourable predictor for drug likeness [26]. Improvement of solubility is possible by attachment of aliphatic polar side chains, which introduce both sp^3^ hybridized carbons and polar groups. However, such side chains can compromise biological activity of the compound if they interfere with the mode of binding to the biological target. In the present study, options for optimization of **1** towards antiplasmodial activity and aqueous solubility were investigated. In this regard, it was essential to identify a position at the molecular structure **1** where side chains are tolerated without loss of biological activity. For the reasons mentioned above it was important to consider the interaction mode of **1** and its congeners with the ATP binding pocket of *Pf*GSK-3. While an X-ray structure of *Pf*GSK-3 has not yet been published, homology models of *Pf*GSK-3 are available which were generated based on *Hs*GSK-3β X-ray structures with different co-crystallized ligands [27]. A docking study utilizing such a homology model predicted the orientation of **1** in the *Pf*GSK-3 binding site as illustrated in Fig. 2 [25]. In this model, the ortho halogen substituent is accommodated in a shallow cavity at the bottom of the ATP binding pocket. The substituents at 5- and 6-position of the parent heterocycle build hydrogen bonds to the hinge area, and the carbonyl oxygen interacts with the conserved lysine (Lys108). The 4-phenyl substituent is directed towards the entrance to the ATP binding pocket, offering its para-position for attachment of additional substituents without hampering the fit to the binding pocket. With a view to challenge this model of inhibitor-enzyme interaction, three series of congeners were synthesized and evaluated for antiplasmodial activity. The congeners carry either small substituents (halogen, methyl, hydroxy; series **2**), alkoxy groups (series **3**), or alkylamino substituents (series **4**) in the designated para position of the 4-phenyl substituent (Fig. 1). In contrast to the target-oriented screening campaign that led to the prototype **1** reported earlier [25], in the present study a phenotypic assay was employed with a view to addressing pharmaceutical properties like membrane permeability in an early development stage. For the determination of antiplasmodial activity, the bioluminescence generated by transgenic NF54-*luc P. falciparum* erythrocytic stages in the presence of test compounds was assessed in a luciferase assay system [28]. The prototype compound **1** and the congeners **2**–**4** display direct single bonds between the thieno[2,3-*b*]pyridine and the phenyl substituent. Due to the ortho chloro-substitution of the phenyl residue the molecules show atropisomerism, and consequently one of the two atropisomers should display stronger biological activity. This hypothesis was corroborated by separation of an active racemic derivative and comparison of the resulting enantiomers regarding enzyme inhibition as well as antiplasmodial activity.

## Methods

### Molecular docking

A homology model of the *Pf*GSK-3 [27] was used for molecular docking. Ligand molecular structures were generated and energy minimized using Molecular Operating Environment (MOE, 2013.08, Chemical Computing Group Inc., Montreal, Canada). Protein and ligand pdb files were saved as mol2 files and loaded utilizing the docking tool GOLD (version 5.2.2.) [30]. To direct the ligands to the ATP binding area, the ligand binding site was defined as a sphere with a radius of 10 Å around the gatekeeper amino acid (Met157). Chemscore [31] with kinase specific parameters implemented in GOLD was applied as scoring function. For the docking, two constraints based on the position of a thieno[2,3-*b*]pyridine in mammalian GSK-3 (pdb entry 3ZDI, [25]) were defined: first, a hydrogen bond between the 6-amino-group of the ligand and a water molecule near the hinge region, and second, a hydrogen bond between the carbonyl oxygen of the ligand and the side chain amino group of the canonical lysine (Lys108). The docking accuracy was defined as 200%, the “generate diverse solutions” option was enabled, and the “early termination” and “save lone pairs” features were disabled. 20 docking runs were performed for each ligand and the resulting poses ranked by score. Visualization of the poses was carried out with PyMol, vers. V0.99 [29]. All poses were visually inspected for plausibility and similarity to the ligand orientation in the template pdb 3ZDI [25].

### Calculation of molecular parameters

The total polar surface area (TPSA) was calculated using the SwissADME interface [32] which utilizes the method developed by Ertl et al. [33].

### Synthesis of test compounds

**Fig. 3:**
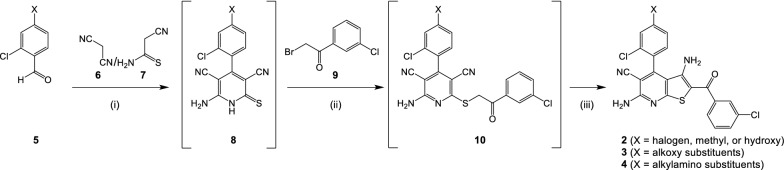
General procedure for preparation of thieno[2,3-*b*]pyridines **2**–**4**. Reagents and conditions: (i) ethanol, piperidine, reflux, 3–6 h; (ii) 10% KOH, DMF, 30 min (iii) 10% KOH, DMF, 1–5 h

The new compounds belonging to series **2**–**4** were synthesized via the synthesis route illustrated in Fig. 3, following published procedures for the synthesis of 3,6-diamino-4-arylthieno[2,3-*b*]pyridin-5-carbonitriles [25, 34–36]. In brief, aromatic aldehydes **5** were reacted with malonodinitrile **6** and cyanothioacetamide **7** in ethanol in the presence of piperidine yielding the 2-thioxo-1,2-dihydropyridines **8**. These intermediates proved to be unstable due to dimerization via oxidation to disulfides and were therefore immediately processed as raw materials. Alkylation of **8** with the 2-bromo-3′-chloroacetophenone **9** in DMF in the presence of potassium carbonate furnished the thioether **10**, which was also not isolated but successively treated with further potassium hydroxide, resulting in a Thorpe-Ziegler ring closure reaction [37] which yielded the desired 3,6-diamino-2-aroyl-4-arylthieno[2,3-*b*]pyridin-5-carbonitriles **2**–**4**. Compound **3f** which exhibits a vicinal dihydroxy motif was prepared from **3e** by acid-catalyzed hydrolytic cleavage of the cyclic acetal function. Compounds **4i** and **4j** were prepared from the Boc-protected precursor molecules **4f** and **4g** by cleavage with trifluoroacetic acid in dichloromethane.

The aromatic aldehydes **5** employed as starting materials for the syntheses of the new thieno[2,3-*b*]pyridines were prepared from either 2-chloro-4-hydroxybenzaldehyde and suitable alkyl halides by means of a Williamson ether synthesis procedure [38] or by nucleophilic substitution of 2-chloro-4-fluorobenzaldehyde with appropriate secondary amines [39].

Molecular structures, details of the syntheses and purification procedures as well as characterization data of all new products are described in the Additional file 1. The structural identity of new test compounds was confirmed by elemental analysis, IR, ^1^H NMR, ^13^C NMR and EI mass spectra. The purity of synthesized products used for biological evaluation was > 95% as determined by HPLC (100% AUC method).

### Separation of enantiomers of compound 4b

The enantiomers of congener **4b** were separated utilizing chiral column chromatography by a commercial separation service lab (Reach Separations, Nottingham, UK). The following separation conditions were employed: column Lux C1, 21.2 mm × 250 mm, 5 µm, flow rate 21 mL/min, detector wavelength 215 nm, injection volume 4000 µL containing 2 mg material, eluent isocratic MeOH/0.1% v/v NH_3_. After chromatographic separation, combined fractions of the purified enantiomers were evaporated to near dryness and transferred into final vessels with dichloromethane. The solvent was removed under a stream of nitrogen at 40 °C before being stored in a vacuum oven at 40 °C and 5 mbar for 16 h to afford the separated enantiomers as yellow solids. The purity of the separated enantiomers was assessed by chiral HPLC. Up to now all efforts to generate single crystals suitable for assignment of the absolute configuration of the separated enantiomers were unsuccessful.

### Luciferase-based viability screening for antiplasmodial activity [28]

For the luciferase-based viability assays, asexual erythrocytic stages of transgenic NF54-*luc P. falciparum* were used. These parasites constitutively express high luciferase levels. The parasites were cultured as described previously [25, 40]. Parasite cultures with parasitaemia of 0.5–1% were dispensed in triplicate into white 96-well flat-bottom plates (each well contains 250 µL) (NUNC, Roskilde, Denmark) and incubated in the presence of 3 µM test compounds for 48 h (37 °C, 90% N_2_, 5% CO_2_, and 5% O_2_). 0.01% DMSO was included in the untreated infected RBC cultures as negative control, since the compounds stock initially was diluted in DMSO and each treatment well also contained 0.01% DMSO. Subsequently, 100 µL RPMI1640 media was removed from each well and a 100 µL volume of the Bright-Glo^®^ substrate solution was added to each well. The resultant cleavage product of the reaction, light, was measured using a FLUOROSKAN FL luminometer (Thermo), to ascertain viable parasites. Untreated cultures were used as negative controls and to calculate the inhibition rate (0% inhibition of parasite growth). Experiments were performed in triplicate and were repeated as a whole at least twice. Blasticidin (Sigma-Aldrich, St. Louis, MO, USA), used for selection of transfected parasites, was included as a positive control on each plate and gave > 90% inhibition of parasite growth at concentration 2 µg/mL. Test compounds exhibiting satisfactorily inhibitory activity (in most cases > 25% inhibition of viability) were rated as actives. For active compounds IC_50_ values were determined from dose–response curves. Calculation of parasite growth inhibition, of the IC_50_ values and statistical analysis were carried out using GraphPad Prism Version 6.0b (GraphPad Software, Inc. San Diego, CA).

### Cytotoxicity assay on HEK293 cells

HEK293T cells were seeded into a solid black flat bottom 96 well plate (2.5 × 10^4^ cells/well) in 200 μL Dulbecco Modified Eagle Medium (DMEM) supplemented with 10% fetal bovine serum and 1% Pen/Strep (final concentration of 100 U/mL Penicillin and 100 μg/mL Streptomycin). Cells were incubated at 37 °C under 5% CO_2_. After 18 h the supernatant was removed and fresh DMEM containing serial dilutions of compounds was added. Compounds were solubilized in DMSO (final DMSO concentration in HEK293T culture was 0.5%.) Wells containing 0.5% DMSO served as a negative control. Plates were incubated for further 48 h at 37 °C under 5% CO_2_. To monitor cell viability the supernatant was removed and cells were incubated with 200 μL 10% PrestoBlue (Invitrogen) in PBS at 37 °C. After 30 min fluorescence (λ_ex_ = 560 nm, λ_em_ = 590 nm) was measured in an EnVision multilable plate reader (Perkin Elmer, integration time 0.1 s/well). Data points were plotted into Graphpad Prism, normalized to the DMSO control and IC_50_ values were calculated using nonlinear regression.

### Production of recombinant *Pf*GSK-3 for Kinase Glo Plus assay

*Pf*GSK-3 was cloned into pOPIN F expression vector [41] using ligation independent cloning (InFusion, Takara Clontech). Expression vector was transformed into *Escherichia coli* C41 and expression of recombinant *Pf*GSK3-6xHis was induced by incubation with 1 mM IPTG at 16 °C overnight. Recombinant protein was purified using immobilized metal affinity chromatography (elution with 250 mM imidazole) and size exclusion chromatography using Superdex200 and 75 16/60 columns (GE Healthcare).

### Kinase-Glo Plus Assay for inhibition of *Pf*GSK-3 and *Hs*GSK-3

Kinase activity was measured using Kinase-Glo Plus Luminescent Kinase Assay (Promega). 20 ng recombinant GSK-3 were incubated for 30 min at 30 °C in the presence of 12 μM GS-1 peptide substrate (YRRAAVPPSPSLSRHSSPHQpSEDEEE, pS stands for phosphorylated serine, Biaffin GmbH & Co KG/proteinkinase.de) and 6 μM ATP (ultra pure, Promega) in a total volume of 5 μL kinase reaction buffer (40 mM Tris/HCl pH 7.5; 20 mM MgCl_2_; 0.1 mg/mL BSA). Kinase reaction mix was transferred to a solid white 384 well plate (NUNC, Thermo Scientific) and kinase reaction was stopped by adding 5 μL RT Kinase-Glo reagent. After 10 min luminescence was measured in an EnVision multilabel plate reader (Perkin Elmer, integration time 0.5 s/well). To investigate inhibition of kinase activity compounds were directly added to the kinase reaction (DMSO concentration did not exceed 1%). All samples were normalized to a negative control (reaction without kinase). Data points were plotted into Graphpad Prism, normalized to a DMSO control and IC_50_ values were calculated using nonlinear regression.

### Radiometric assay for inhibition of *Pf*GSK-3

Recombinant GST-*Pf*GSK-His was expressed in Rosetta 2 (DE3) cells and purified on cobalt affinity resin as described previously [19]. Recombinant *Pf*GSK-3 (0.25 mg) was assayed with 48 µM GS-1 peptide as a substrate, in 25 mM Tris–HCl pH 7.5, 10 mM MgCl_2_, 1 mM EGTA, 1 mM EDTA, 0.5 mg/mL BSA, 5 mM DTT, 2 µg/mL heparin, in the presence of 15 µM [γ-^32^P] ATP (3000 Ci/mmol; 1 mCi/mL) (Perkin-Elmer, USA) in a final volume of 25 µL. After 30 min incubation at 30 °C, 8 µL aliquots were spotted onto 1 × 1 cm pieces of P81 phosphocellulose paper (Reaction Biology, Malvern, PA, USA) and 20 s later, the filters were washed five times (for at least 5 min each time) in a solution of 10 mL phosphoric acid/L water. Filters were rinsed in acetone and the dried filters and radioactivity was measured by Cherenkov counting. Blank values were subtracted and activities were expressed in percentage of the maximal activity, i.e. in the absence of inhibitors. Controls were performed with appropriate dilutions of dimethylsulfoxide.

### Determination of kinetic solubility

Kinetic solubility indicates the solubility of the most rapidly precipitating form. Kinetic solubilities of **1** and **4h** were determined by laser nephelometry [42, 43] using the NEPHELOstarPlus nephelometer (BMG LabTech, Ortenberg, Germany) and F-bottom transparent 96-well plates. The instrument uses red laser light (635 nm, 80% intensity), which passes through the 96-well plate and which is scattered by turbid suspensions. Each compound was measured six times at 25 °C by preparing three independent stock solutions in concentrations of 5 to 10 mM in DMSO. From each stock solution two independent dilution series were prepared. Based on preliminary experiments, dilution concentrations were chosen such that at least five to six concentrations below and above the expected point of precipitation were recorded. To the 96-well plate containing 198 µL aqueous phosphate buffer at pH 7.4, 2 µL of each dilution sample in DMSO was added and shaken for 10 s. at 700 rpm (double orbital). Phosphate buffer pH 7.4 containing sodium chloride was prepared by dissolving disodium monohydrogen phosphate-dodecahydrate (2.38 g), potassium dihydrogen phosphate (0.19 g) and sodium chloride (8.0 g) in water to a volume of 1000.0 mL. The pH was adjusted by adding sulfuric acid. First, the plates were scanned without content to quantify the background, which was later subtracted from the obtained values of each individual well. As blank, 2 µL of neat DMSO was added to the phosphate buffer. Close to the point of precipitation, particle concentration can be assumed to be proportional to the intensity of scattered light. The solubility curve is typically characterized by a distinct kick-off point. The concentration at the precipitation point corresponds to the intersection of two regression curves that result from separate analyses of dissolved and turbid samples. The calculations were performed with Microsoft Excel 2013.

## Results

Considering the current resistance situation against approved anti-malarial drugs, new anti-plasmodial compounds acting by hitherto unexploited mechanisms are urgently required. The study reported here was aimed at identifying structural options for the optimization of the known *Pf*GSK-3 inhibitor **1**. First, it was addressed which position in the molecule is suitable for the attachment of side chains without losing the biological activity. Second, it was investigated whether the enantiomers of active compounds show differing activity, an assumption deduced from the orientation of the inhibitors in the ATP binding pocket of *Pf*GSK-3 predicted by molecular docking studies. In contrast to the target-oriented screening which initially identified the thieno[2,3-*b*]pyridines as *Pf*GSK-3 inhibitors [25], in the present study a phenotypic assay was utilized with the aim of taking into account the membrane permeability of the inhibitor molecules at an early stage of development. Based on the putative orientation of **1** in the ATP-binding site of *Pf*GSK-3, the para position of the 4-phenyl substituent was selected as suitable attachment point for side chains. The compounds that were synthesized as novel congeners carry at the indicated position either small substituents (halogen, methyl, hydroxy; series **2**), alkoxy groups (series **3**), or alkylamino substituents (series **4**) (Fig. 1). The results of biological activity testing showed that a fluoro- or a chloro-substituent at the indicated para position of the 4-phenyl ring were tolerated without loss of antiplasmodial activity (**2a**,**b**), whereas larger substituents (bromo-or methyl, **2c**,**d**) or a phenolic hydroxyl group (**2e**) decreased the antiparasitic properties. Organic substituents attached via an oxygen atom (series **3**) generally led to diminished or completely lost antiplasmodial activity compared to prototype **1**. Whether this observation is based on insufficient *Pf*GSK-3 inhibitory activity by congeners **3** or because of poor permeation through erythrocyte and parasite membranes is not yet clear. The derivatives **4** in which monoamines (dimethylamino, pyrrolidino, piperidino or morpholino) are attached via the nitrogen atom were equipotent to **1**. Evaluation of the separated enantiomers of the diethylamino derivate **4b** revealed that the (−) enantiomer completely failed to inhibit the parasites, while the (+) enantiomer led to improved inhibitory activity. The derivatives with the highest antiplasmodial activity in the series were represented by **4h** (the dimethylaminoethyl derivative) and **4j** (the piperazine derivative) which both contain an additional basic nitrogen in the attached side chain. A noticeable exception is **4i**, the only derivative bearing a primary basic aliphatic amino group in its side chain. Supposedly **4i** is unable to permeate through biological membranes due to its higher total polar surface area (TPSA) compared to the closely related tertiary amine **4h** (**4i**: TPSA 163.3 Å^2^; **4h**: TPSA 140.5 Å^2^).

Considering the antiplasmodial activity of all new compounds reported here, congener **4h** appeared as particularly promising, exhibiting more than fourfold higher antiplasmodial potency versus the prototype compound **1**. In this respect it was of interest also to compare the kinase inhibitory activity and the solubility of optimized structure **4h** and prototype **1** (Table 1). Both in a Kinase Glo Plus assay and in a radiometric assay, **1** and **4h** produced submicromolar *Pf*GSK-3 inhibition values. In both assay settings, **1** proved to be more potent than **4h**, indicating that the higher antiplasmodial activity of **4h** might be the result of pharmacokinetic features like cellular uptake, efflux or metabolism. In the Kinase Glo Plus assay it was also shown that both **1** and **4h** selectively inhibit the plasmodial versus the human GSK-3 orthologue with selectivity indices of 38 and 56, respectively. Although in the Kinase Glo Plus assay a lower ATP concentration (6 µM) was used than in the radiometric assay (15 µM), both **1** and **4h** showed lower IC_50_ values in the latter test system. While a conclusive explanation for this observation is not yet available, these results show that a comparison of IC_50_ values is pertinent only for data generated in the same test system. Viability tests with HEK293T cells versus plasmodial *Pf*NF54-Luc erythrocytic stage cells revealed similar selectivity indices for **1** and **4h** (SI = 6.4 and 4.5, respectively). Determination of kinetic solubility by laser nephelometry [42, 43] showed that **4h** displays more than threefold higher solubility (S_0 *p*H7.4_ = 4.8 µM) compared to **1** (S_0 *p*H7.4_ = 1.5 µM) (Table 2).

**Table 1:** Results of biological evaluation

Code	X–^a^	% inhibition ± SD, *Pf*NF54-Luc at 3 µM	IC_50_ [µM], *Pf*NF54-Luc
**1** [25]	H–	n.d.	5.5
**2a**	Cl–	25.7 ± 3.5	6.2
**2b**	F–	28.1 ± 3.3	6.1
**2c**	Br–	− 5.9 ± 1.3	n.d.
**2d**	H_3_C–	12.4 ± 2.4	n.d.
**2e**	HO–	0.2 ± 2.5	n.d.
**3a**	H_3_CO–	33.2 ± 3.2	12.2
**3b**	H_5_C_2_O–	14.5 ± 1.3	n.d.
**3c**	C_6_H_5_–CH_2_–O–	12.8 ± 4.9	n.d.
**3d**	2-hydroxyethoxy–	25.6 ± 3.3	20.0
**3e**	(2,2-dimethyl-1,3-dioxolan-4-yl)methoxy–	− 11.7 ± 0.7	n.d.
**3f**	2,3-dihydroxypropoxy–	14.7 ± 1.8	n.d.
**3g**	HO_2_C–CH_2_–O	31.3 ± 1.1	n.d.
**4a**	Dimethylamino–	29.9 ± 5.6	5.7
**(+)4b**	Diethylamino–	73.0 ± 1.2	1.1
**(−)4b**	Diethylamino–	− 24.4 ± 10.4	n.d.
**4c**	Pyrrolidino–	33.0 ± 2.4	n.d.
**4d**	Piperidino–	39.1 ± 1.1	5.0
**4e**	Morpholino–	20.2 ± 4.8	n.d.
**4f**	*N*-[2-(*N*-Boc-amino)ethyl)]-*N*-(methyl)amino–	67.1 ± 2.7	2.5
**4g**	*N*^4^-Boc-piperazino–	42.1 ± 2.9	3.8
**4h**	*N*-(2-dimethylaminoethyl)-*N*-(methyl)amino–	99.9 ± 0.1	1.2
**4i**	*N*-(2-aminoethyl)-*N*-(methyl)amino-(as hydrochloride)	8.1 ± 3.3	n.d.
**4j**	Piperazino-(as hydrochloride)	75.7 ± 1.5	1.5
BS	Blasticidin	99.3 ± 0.7	n.d.

*n.d.* not determined

^a^for position of X, refer to Fig. 3. All structures are depicted in the Additional file 1

**Table 2:** Comparison of biological, structural and physicochemical properties of improved congener **4h** and prototype **1**

ID	IC_50_ [µM], *Pf*GSK-3^a^ (Kinase Glo Plus)	IC_50_ [µM], *Hs*GSK-3^a^ (Kinase Glo Plus)	IC_50_ [µM], *Pf*GSK-3^b^ (radiometric)	IC_50_ [µM], *Pf*NF54-Luc	IC_50_ [µM], HEK 293T^c^	SI^d^	TPSA [Å^2^]^e^	Fsp^3 f^	S_0 p H 7.4, exp_ [µM]^g^
**1**	0.24 (0.21–0.27)	9.08 (6.72–12.5)	0.151 (0.154–0.148)	5.5	35.2 (27.0–46.3)	6.4	134.0	0.00	1.5 ± 0.3
**4h**	0.72 (0.68–0.75)	40.2 (38.3–42.2)	0.184 (0.179–0.190)	1.2	5.56 (5.05–6.03)	4.6	140.5	0.19	4.8 ± 0.9

^a^Kinase Glo Plus assay, mean value of three determinations, range given in brackets

^b^Radiometric assay, mean value of two determinations, range given in brackets

^c^HEK293T cell viability assay, mean value of six determinations, range given in brackets

^d^SI (selectivity index) = IC_50_ HEK293T/IC_50_ *Pf*NF54-Luc

^e^Calculation of TPSA according to Ertl et al. [33] through Swiss ADME [32]

^f^Number of sp^3^ hybridized carbons/total carbon count [26]

^g^Kinetic solubility determined by nephelometry in aqueous buffer (pH 7.4) in the presence of 1% DMSO [42, 43]; mean ± standard deviation

**Fig. 4:**
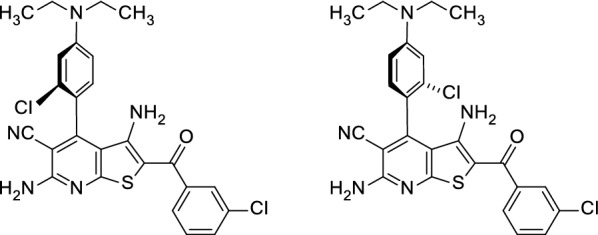
Enantiomers of thieno[2,3-*b*]pyridine **4b**. The rotation around the biaryl bond connecting the heterocycle and the 4-phenyl substituent is hindered by the ortho chloro substituent at the phenyl ring and the ortho cyano substituent in combination with the thieno anneland at the pyridine ring

The prototype compound **1** and the congeners **2**–**4** display direct single bonds between the thieno[2,3-*b*]pyridine and the phenyl substituent. Due to the ortho chloro-substitution of the latter the free rotation around the biaryl linkage is restricted, resulting in axial chirality of the molecules (Fig. 4). It has been shown previously that a double substitution with chlorine at both ortho positions leads to decreased *Pf*GSK-3 inhibition [44]. If the orientation as illustrated for **1** in Fig. 2 is realistic, only the R_a_-configured enantiomers can be accommodated to the ATP-binding site, because in this binding mode only the R_a_ enantiomer is able to fit the ortho chloro substituent into place without causing a spatial clash. In consequence, the R_a_ enantiomer should act as eutomer, displaying distinctly stronger *Pf*GSK-3 inhibition and antiplasmodial activity. To test this assumption, the enantiomers of one representative (**4b**) of the 4-(alkylamino)phenyl series were separated by chromatography on a chiral column. Indeed, only the enantiomer (+)-**4b** exhibited antiplasmodial activity and was much more potent as *Pf*GSK-3 inhibitor than the (−)-**4b** enantiomer (*Pf*GSK-3 IC_50_-values in the Kinase Glo Plus assay: (+)-**4b**: 39.6 (35.1–45.5) µM; (−)-**4b**: > 100 µM). Both enantiomers did not show any inhibitory activity on the human GSK-3 orthologue (*Hs*GSK-3 IC_50_-values in the Kinase Glo Plus assay: (+)-**4b**, (−)-**4b**: > 100 µM). So far, no single crystals of (+)-**4b** or (−)-**4b** could be generated, so that an unambiguous assignment of the configuration of these enantiomers on the basis of X-ray structure analyses was impossible up to now.

## Discussion

The 4-phenylthieno[2,3-*b*]pyridine **1** acts as antiplasmodial agent presumably due to its selective *Pf*GSK-3 inhibitory activity. For optimization of potency and aqueous solubility molecular modifications of **1** compliant with its binding mode to the molecular target are necessary. In this regard, the para position at the 4-phenyl substituent in structure **1** was identified as suitable attachment point for side chains. However, the nature of the link between the side chain and the parent scaffold was crucial for the properties of the derived congeners. Whereas alkoxy groups diminished the antiplasmodial activity, alkylamino chains tended to improve this property. As most promising result of the study, derivative **4h** shows better aqueous solubility, retained *Pf*GSK-3 inhibitory activity, improved antiplasmodial potency as well as selectivity versus human cells. However, further optimization in this class is necessary before the molecules may be assumed as lead structures for a preclinical development. For this optimization process, the structure activity relationships disclosed here will be valuable. At the same time it was shown that the axial chirality displayed by the title compounds is of paramount importance for biological activity, an interesting feature which has been discussed for implications in drug development [45, 46], and has been exploited for enhancing the selectivity of kinase inhibitors [47]. In further optimization campaigns it has to be taken into account that not a racemate, but a separated and purified eutomer will be the drug compound to be evaluated and developed.

## Conclusions

The attachment of alkylamino side chains leads to the improvement of antiplasmodial activity and aqueous solubility of *Pf*GSK-inhibitors belonging to the class of 4-phenylthieno[2,3-*b*]pyridines. These molecules show axial chirality, a feature of high impact for biological activity. The findings can be exploited for the development of improved selective *Pf*GSK-3 inhibitors. Such studies should comprise further broad modifications of the aminoalkyl side chains as well as separation and application of the active eutomer in biological test systems.

## Additional file

**Additional file 1.** Molecular structures, details of the syntheses and purification procedures as well as characterization data of all new products.

## Abbreviations

ACT: artemisinin-based combination therapy
ADME: absorption, distribution, metabolism, excretion
AMA1: apical membrane antigen 1
AUC: area under the curve
DMEM: Dulbecco Modified Eagle Medium
DMF: *N*,*N*-dimethylformamide
DMSO: dimethyl sulfoxide
EI: electron impact
Fsp^3^: fraction of sp^3^ hybridized carbon atoms
GS: glycogen synthase
HEK: human embryonic kidney
HPLC: high performance liquid chromatography
*Hs*GSK-3: *Homo sapiens* glycogen synthase kinase-3
IC_50_: concentration for 50% inhibition
IPTG: isopropyl β-d-1-thiogalactopyranoside
IR: infra-red
NMR: nuclear magnetic resonance
PCR: polymerase chain reaction
*Pf*GSK-3: *Plasmodium falciparum* glycogen synthase kinase-3
RBC: red blood cell
RPMI media: Roswell Park Memorial Institute media
TPSA: topological polar surface area
WHO: World Health Organization
