# Supplementary material for: Structure–activity relationships in a series of antiplasmodial thieno[2,3-b]pyridines
Source: Malar J. 2019 Mar 21;18:89. doi: 10.1186/s12936-019-2725-y (PMC6429710; doi:10.1186/s12936-019-2725-y)
Supplement: Supplementary file 1 — Additional file 1. Molecular structures, details of the syntheses and purification procedures as well as characterization data of all new products. [file 12936_2019_2725_MOESM1_ESM.pdf]

Andreas Masch, Abed Nasereddin, Arne Alder, Megan J. Bird, Sandra I. Schweda, Lutz Preu, Christian Doerig, Ron Dzikowski, Tim W. Gilberger, Conrad Kunick

Structure-activity relationships in a series of antiplasmodial thieno[2,3-*b*]pyridines

Additional file: Molecular structures, details of the syntheses and purification procedures as well as characterization data of all new products

Table of Contents

1. Apparatus and Materials 2

2. General Procedures 3

3. Syntheses of 3,6-Diamino-4-aryl-2-(3-chlorobenzoyl)thieno[2,3-*b*]pyridine-5-carbonitriles 5

4. Syntheses of Substituted Benzaldehydes 28

5. Syntheses of 6-Amino-4-aryl-2-thioxo-1,2-dihydropyridine-3,5-dicarbonitriles 36

6. Synthesis of a 3-Aryl-2-cyanoprop-2-enthioamide 45

1. Apparatus and Materials

Starting materials for syntheses were purchased from the following suppliers if not indicated otherwise: Acros Organics (Geel, Belgium), Sigma Aldrich (Steinheim, Germany), Alfa Aesar /Thermo Fisher (Kandel) (Karlsruhe, Germany) and were used without further purification. 2-Chlorobenzaldehydes were purchased from Activate Scientific (Prien, Germany). Solvents were used without prior drying procedures, if not indicated otherwise. Dichloromethane was dried by storing over anhydrous P_2O_5 and subsequent refluxing and distillation from P_2O_5. Dried dichloromethane was stored over molecular sieves (3Å). Melting points were determined on an electric variable heater (Barnstead Electrothermal IA 9100, Electrothermal Engineering, Southend-on-Sea, UK) in open glass capillaries and are uncorrected. IR spectra were recorded as KBr disks on a Thermo Nicolet FT-IR 200 (Thermo Nicolet, Madison, WI, USA). ^{1}H-NMR spectra and ^{13}C-NMR spectra: Bruker Avance DRX-400, Bruker Avance III-400, Bruker Avance II-600 (Bruker Corporation, Billerica, MA, USA) recorded at the NMR laboratories of the Chemical Institutes of the Technische Universität Braunschweig. Spectra were evaluated using MestReNova version 8.1.2-11880 (Mestrelab Research S.L. 2013, Escondido, CA, USA). Chemical shifts were recorded as δ values in ppm and are referenced to tetramethylsilane as internal standard. Signals in ^{13}C spectra were assigned based on the result of ^{13}C DEPT135 experiments. Elemental analyses were determined on a CE Instruments FlashEA 1112 elemental analyzer (Thermo Quest, San Jose, CA, USA). Mass spectra were recorded on a Finnigan-MAT 95 (Thermo Finnigan MAT, Bremen, Germany). Accurate measurements were conducted according to the peak match method using perfluorokerosene (PFK) as an internal mass reference. (EI) MS: ionization energy 70 eV. (ESI)-MS: Samples were diluted to a concentration of 50 µg/mL in MeOH with addition of tetradecyltrimethylammonium bromide (0.1 mg/mL) and were injected directly. Spraying potential in positive mode 2.3–2.8 kV (Department of Mass Spectrometry of the Chemical Institutes of the Technische Universität Braunschweig). TLC: Polygram Sil G/UV_{254} (Macherey-Nagel, Düren, Germany), 40 mm × 80 mm, visualization by UV illumination (254 and 366 nm), eluents: ethyl acetate, toluene, petrol ether, triethylamine, formic acid, and mixtures of these solvents. Purity was determined by HPLC using isocratic and gradient elution performed on Merck Hitachi Elite LaChrom systems (Hitachi High Technologies Inc., San Jose, CA, USA). Isocratic elution: LaChrom Elite pump L-2130, autosampler L-2200, column oven L-

2300 (40 °C), diode-array-detector L-2450, organizer box L-2000. Gradient elution: LaChrom Elite pump L-2130, autosampler L-2200, UV-detector L-2400, organizer box L-2000; column: Merck LiChroCART 125-4, LiChrospher 100 RP-18, 5 µM (Merck, Darmstadt, Germany). Sample preparation: 0.2-0.3 mg were dissolved in 300 µL DMSO. The solution was filtered through a Whatman Mini-UniPrep filter. Injection volume 5–10 µL; flow rate 1.000 mL/min; integration cut off 0–1.55 min, threshold 1000; run time for isocratic elution 15 min; dead time (t_m) related to DMSO signal; gross retention time (t_{ms}); time program for gradient elution 0–2 min: ACN/H_2O 10:90, 2–12 min: ACN/H_2O 10:90 → 90:10, 12–20 min: ACN/H_2O 90:10; data evaluation by by 100% AUC method (EZ Chrom Elite Client/Server Version 3.1.3). Absorbance maxima (λ_{max}) were extracted from DAD spectra. Buffer for isocratic elution: triethylamine (20 mL) and sodium hydroxide (242 mg) were dissolved in water up to a volume of 1 L. Sulfuric acid was added to adjust to pH 2.7 (632 pH-meter, Metrohm, Filderstadt, Germany). Polarimetry: MCP 100 (Anton Paar GmbH, Ostfildern-Scharnhausen, Germany), wavelength 589 nm, range of evaluation ±89.9 °, cell for measurement 100 mm; sample preparation: 40.0 mg of test compound were dissolved in dichloromethane (10 mL) and evaluated at 20 °C.

2. General Procedures

General procedure 1 (GP 1): Syntheses of 4-alkoxy-2-chlorobenzaldehydes

The indicated alkyl halide (51.0 mmol) is added to a a solution of 2-chloro-4-hydroxybenzaldehyde (7.00 g, 51.0 mmol) and potassium carbonate (14.2 g, 102 mmol) in the indicated solvent. After refluxing for 6–24 h the mixture is evaporated. The resulting residue is suspended in aqueous sodium hydroxide solution (25%) and extracted with ethyl acetate (100 mL). After evaporation of the organic layer, the oily residue is blended with silica gel (3 g) and ethyl acetate (20 mL), evaporated, and subsequently eluted with the indicated solvent by means of column chromatography.

General procedure 2 (GP 2): Syntheses of 4-(*N*,*N*-dialkylamino)-2-chloro-benzaldehydes

An appropriate secondary amine (7.50 mmol) is added to a mixture of 2-chloro-4-fluorobenzaldehyde (793 mg, 5.00 mmol) and potassium carbonate (1.11 g, 8.00 mmol) in DMF (15 mL). After stirring at 100 °C for 5-20 h, the mixture is poured

on ice water (20 g). A yellow precipitate is formed, which is filtered off with suction and subsequently purified either by crystallization from water or by column chromatography.

General procedure 3 (GP 3): Syntheses of 6-amino-4-aryl-2-thioxo-1,2-dihydropyridine-3,5-dicarbonitriles

Piperidine (one drop) is added to a slurry of malonodinitrile (66.1 mg, 1.00 mmol), 2-cyanothioacetamide (100 mg, 1.00 mmol) and an appropriate para substituted 2-chlorobenzaldehyde (1.00 mmol) in ethanol (4 mL). After refluxing for 3-6 h, the mixture is evaporated. To the oily residue are added water (10 mL), dichloromethane (2 mL), and acetic acid (6 drops). Upon storage in the fridge at 2-5 °C, a brown precipitate is formed, which is either purified as indicated or used without further purification for the following synthesis step.

General procedure 4 (GP 4): Syntheses of 3,6-diamino-2-aroyl-4-arylthieno[2,3-*b*]pyridine-5-carbonitriles

An aqueous solution of potassium hydroxide (10%, 224 µL, 0.40 mmol) is added dropwise to a solution of an approriate 4-aryl-2-thioxo-1,2-dihydropyridine (0.40 mmol) in DMF (0.5 mL). After one minute of stirring, the appropriate phenacyl bromide (0.40 mmol) is added. After further stirring for 30 min, a second portion of aqueous potassium hydroxide solution (10%, 224 µL, 0.40 mmol) is added. Stirring is continued until the open chain thioether intermediate is no longer detectable by tlc. Water (5 mL) is added, and the resulting precipitate is filtered off and washed thoroughly with water. The material is purified by column chomatography and subsequent crystallization.

General procedure 5 (GP 5): Cleavage of protecting groups (Boc-groups or *tert*-butyl esters)

The appropriate protected educt (1.00 mmol) is dissolved in dry dichloromethane (7 mL). Trifluoroacetic acid (3.5 mL) is added and the mixture is stirred for 12–17 h under nitrogen. Subsequently the mixture is evaporated. The remaining oily residue is dissolved in propan-2-ol (4 mL). A 2 M solution of hydrogen chloride in propan-2-ol

(12 drops) is added. Upon addition of diethyl ether (20 mL) a yellow solid precipitates. After refluxing for further 1.5 h, the mixture is allowed to cool to room temperature. The resulting precipitate is filtered off with suction.

3. Syntheses of 3,6-Diamino-4-aryl-2-(3-chlorobenzoyl)thieno[2,3-*b*]pyridine-5-carbonitriles

3,6-Diamino-2-(3-chlorobenzoyl)-4-(2,4-dichlorophenyl)thieno[2,3-*b*]pyridine-5-carbonitrile (**2a**)

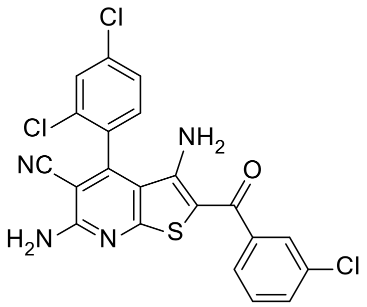

Prepared following GP 4 from 6-amino-4-(2,4-dichlorophenyl)-2-thioxo-1,2-dihydropyridine-3,5-dicarbonitrile (80.0 mg, 0.249 mmol), potassium hydroxide solution (10%) (twice 138 µL, 0.492 mmol) and 2-bromo-3'-chloroacetophenone (**9**, 58.2 mg, 0.249 mmol). Reaction time: 2 h. Crystallization from ethanol (70% v/v) yielded 52 mg (55%) yellow powder.

Mp: 257–259 °C; IR (KBr): 3481 cm^{-1} and 3347 cm^{-1} (NH), 2217 cm^{-1} (C≡N), 1613 cm^{-1} (C=O); ^{1}H-NMR (DMSO-d_6, 600.1 MHz): δ (ppm) = 6.83 (br s, 2H, NH_2), 7.54–7.57 (m, 1H, ArH), 7.62–7.64 (m, 1H, ArH), 7.64–7.68 (m, 2H, ArH), 7.70 (d, 1H, J = 8.3 Hz, ArH), 7.74 (dd, 1H, J = 8.2/2.0 Hz, ArH), 7.83 (br s, 2H, NH_2), 8.02 (d, 1H, J = 2.0 Hz, ArH); ^{13}C-NMR (DMSO-d_6, 150.9 MHz): δ (ppm) = 125.5, 126.7, 128.7, 130.0, 130.4, 130.6, 131.2 (CH); 90.7, 99.5, 112.3, 114.6, 130.6, 132.2, 133.2, 136.1, 142.5, 149.7, 151.2, 159.1, 166.4, 185.8 (C); $C_{21}H_{11}Cl_3N_4OS$ (473.76); calcd C 53.24, H 2.34, N 11.83; found C 53.14, H 2.49, N 11.43; MS (EI): m/z (%) = 473.0 $[M]^{+\bullet}$ (100), 437.0 $[M^{+\bullet}$–Cl] (26); isocrat. HPLC: 99.3% at 254 nm and 98.4% at 280 nm, t_{ms} = 8.10 min, t_m = 1.12 min (ACN/H_2O 60:40); λ_{max} (nm): 324, 225, 297; gradient HPLC: 98.6% at 254 nm, t_{ms} = 13.7 min, t_m = 1.22 min (0–2 min: ACN/H_2O 10:90, 2-12 min: ACN/H_2O 10:90 → 90:10, 12–20 min: ACN/H_2O 90:10).

3,6-Diamino-2-(3-chlorobenzoyl)-4-(2-chloro-4-fluorophenyl)thieno[2,3-*b*]pyridine-5-carbonitrile (**2b**)

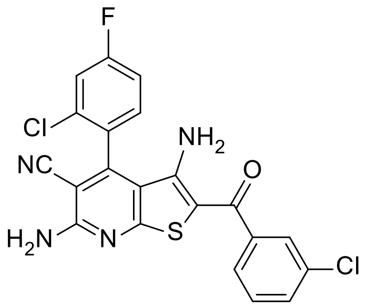

Prepared following GP 4 from 6-amino-4-(2-chloro-4-fluorophenyl)-2-thioxo-1,2-dihydropyridine-3,5-dicarbonitrile (515 mg, 1.71 mmol), potassium hydroxide solution (10%) (twice 950 µL, 1.70 mmol) and 2-bromo-3'-chloraocetophenone (**9**, 395 mg, 1.69 mmol). Reaction time: 90 min. Crystallization from ethanol (70% v/v) yielded 775 mg (62%) yellow powder.

Mp: 247–250 °C; IR (KBr): 3476 cm^{-1} and 3393 cm^{-1} (NH), 2213 cm^{-1} (C≡N), 1604 cm^{-1} (C=O); ^{1}H-NMR (DMSO-d_6, 600.1 MHz): δ (ppm) = 6.88 (br s, 2H, NH_2), 7.53–7.58 (m, 2H, ArH), 7.60–7.69 (m, 3H, ArH), 7.71–7.76 (m, 1H, ArH), 7.82 (br s, 2H, NH_2), 8.84–7.87 (m, 1H, ArH); ^{13}C-NMR (DMSO-d_6, 150.9 MHz): δ (ppm) = 115.9 (d, $^{2}J_{C,F}$ = 21.6 Hz), 117.9 (d, $^{2}J_{C,F}$ = 25.8 Hz), 125.5, 126.8, 130.5, 130.7, 131.7 (d, $^{3}J_{C,F}$ = 9.24 Hz) (CH); 91.1, 99.5, 112.4, 114.7, 128.1 (d, $^{4}J_{C,F}$ = 3.4 Hz), 132.3 (d, $^{3}J_{C,F}$ = 11.1 Hz), 133.2, 142.6, 150.0, 151.2, 159.2, 162.1 (d, $^{1}J_{C,F}$ = 251 Hz), 166.3, 185.8 (C); $C_{21}H_{11}Cl_2FN_4OS$ (456.00); calcd C 55.16, H 2.42, N 12.25; found C 54.98, H 2.30, N 11.86; MS (EI): m/z (%) = 455.0 $[M]^{+\bullet}$ (100), 421.0 $[M^{+\bullet}$–Cl] (24); isocrat. HPLC: 99.7% at 254 nm and 99.6% at 280 nm, t_{ms} = 5.91 min, t_m = 1.2 min (ACN/H_2O 60:40); λ_{max} (nm): 328, 294; gradient HPLC: 95.4% at 254 nm, t_{ms} = 13.2 min, t_m = 1.25 min (0–2 min: ACN/H_2O 10:90, 2–12 min: ACN/H_2O 10:90 → 90:10, 12–20 min: ACN/H_2O 90:10).

<u>3,6-Diamino-4-(4-bromo-2-chlorophenyl)-2-(3-chlorobenzoyl)thieno[2,3-*b*]pyridine-5-carbonitrile (**2c**)</u>

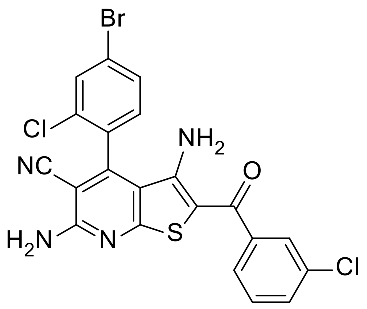

Prepared following GP 4 from 6-amino-4-(4-bromo-2-chlorophenyl)-2-thioxo-1,2-dihydropyridine-3,5-dicarbonitrile (450 mg, 1.23 mmol), potassium hydroxide solution (10%) (twice 689 µL, 1.23 mmol) and 2-bromo-3'-chloracetophenone (**9**, 283 mg, 1.23 mmol). Reaction time: 60 min. Crystallization from ethanol (70% v/v) yielded 227 mg (36%) yellow powder.

Mp: 324–330 °C (dec.); IR (KBr): 3481 cm^{-1} and 3343 cm^{-1} (NH), 2218 cm^{-1} (C≡N), 1613 cm^{-1} (C=O); ^{1}H-NMR (DMSO-d_6, 600.1 MHz): δ (ppm) = 6.83 (br s, 2H, NH_2), 7.53–7.57 (m, 1H, ArH), 7.61–7.64 (m, 2H, ArH), 7.64–7.68 (m, 2H, ArH), 7.83 (br s, 2H, NH_2), 7.86–7.88 (m, 1H, ArH), 8.11–8.14 (m, 1H, ArH); ^{13}C-NMR (DMSO-d_6, 150.9 MHz): δ (ppm) = 125.5, 126.7, 130.4, 130.7, 131.4, 131.6, 132.7 (CH); 90.7, 99.5, 112.2, 114.7, 124.7, 131.0, 132.3, 133.3, 142.6, 149.8, 151.2, 159.1, 166.4, 185.8 (C); $C_{21}H_{11}BrCl_2N_4OS$ (517.92); calcd C 48.67, H 2.14, N 10.81; found C 48.72, H 2.05, N 10.58; MS (EI): m/z (%) = 516.9 $[M]^{+\bullet}$ (100); isocrat. HPLC: 99.0% at 254 nm and 99.5% at 280 nm, t_{ms} = 8.49 min, t_m = 1.2 min (ACN/H_2O 60:40); λ_{max} (nm): 328, 296; gradient HPLC: 97.6% at 254 nm, t_{ms} = 13.9 min, t_m = 1.25 min (0-2 min: ACN/H_2O 10:90, 2–12 min: ACN/H_2O 10:90 → 90:10, 12–20 min: ACN/H_2O 90:10).

<u>3,6-Diamino-2-(3-chlorobenzoyl)-4-(2-chloro-4-methylphenyl)thieno[2,3-*b*]pyridine-5-carbonitrile (**2d**)</u>

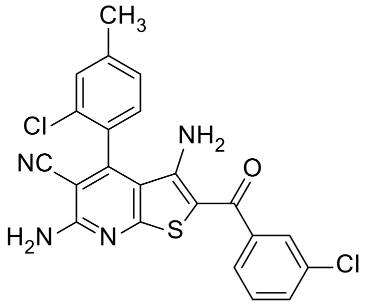

Prepared following GP 4 from 6-amino-4-(2-chloro-4-methylphenyl)-2-thioxo-1,2-dihydropyridine-3,5-dicarbonitrile (306 mg, 1.01 mmol), potassium hydroxide solution (10%) (twice 566 µL, 1.01 mmol) and 2-bromo-3'-chloroacetophenone (**9**, 240 mg, 1.03 mmol); reaction time 60 min. Crystallization from ethanol (70% v/v) yielded 289 mg (63%) yellow powder.

Mp: 342–348 °C (dec.); IR (KBr): 3475 cm^{-1}, 3340 cm^{-1} and 3210 cm^{-1} (NH), 2217 cm^{-1} (C≡N), 1612 cm^{-1} (C=O); ^{1}H-NMR (DMSO-d_6, 600.1 MHz): δ (ppm) = 2.46 (s, 3H, CH_3), 6.59 (br s, 2H, NH_2), 7.43–7.47 (m, 1H, ArH), 7.49–7.58 (m, 2H, ArH), 7.60–7.69 (m, 4H, ArH), 7.78 (br s, 2H, NH_2); ^{13}C-NMR (DMSO-d_6, 150.9 MHz): δ (ppm) = 20.6 (CH_3); 125.5, 126.8, 129.1, 129.5, 130.5, 130.6, 130.6 (CH); 91.0, 99.5, 112.4, 114.8, 128.7, 130.7, 133.3, 142.5, 142.6, 150.9, 151.2, 159.2, 166.3, 185.7 (C); $C_{22}H_{14}Cl_2N_4OS$ (452.03); calcd C 58.29, H 3.11, N 12.36; found C 57.99, H 3.07, N 11.96; MS (EI): m/z (%) = 452.0 $[M]^{+\bullet}$ (100), 417.0 $[M^{+\bullet}$–Cl] (52); isocrat. HPLC: 97.1% at 254 nm and 97.9% at 280 nm, t_{ms} = 7.88 min, t_m = 1.2 min (ACN/H_2O 60:40); λ_{max} (nm): 327, 296; gradient HPLC: 98.5% at 254 nm, t_{ms} = 13.7 min, t_m = 1.25 min (0–2 min: ACN/H_2O 10:90, 2–12 min: ACN/H_2O 10:90 → 90:10, 12–20 min: ACN/H_2O 90:10).

3,6-Diamino-2-(3-chlorobenzoyl)-4-(2-chloro-4-hydroxyphenyl)thieno[2,3-*b*]pyridine-5-carbonitrile (**2e**)

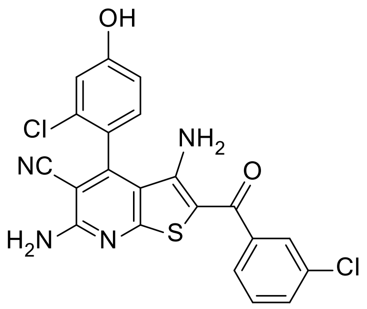

To a solution of 3,6-diamino-2-(3-chlorobenzoyl)-4-(2-chloro-4-methoxyphenyl)thieno[2,3-*b*]pyridine-5-carbonitrile (186 mg, 0.397 mmol) in dry dichloromethane (5 mL) was added boron tribromide (1.5 mL) with stirring. Stirring was continued for 12 h at room temperature. Water (10 mL) was added and stirring was continued for further 2 h. The resulting precipitate was filtered off with suction and washed with water. Purification by column chromatography (ethyl acetate/petrol ether 1:1) yielded 80.0 mg (45%) of a yellow powder.

Mp: 214–218 °C (dec.); IR (KBr): 3477 cm^{-1} and 3307 cm^{-1} (NH), 2218 cm^{-1} (C≡N), 1637 cm^{-1} (C=O); ^{1}H-NMR (DMSO-d_6, 600.1 MHz): δ (ppm) = 6.77 (br s, 2H, NH_2), 7.00 (dd, J = 8.4/2.4 Hz, 1H, ArH), 7.12 (d, J = 2.3 Hz, 1H, ArH), 7.42 (d, J = 8.4 Hz, 1H, ArH), 7.51–7.58 (m, 1H, ArH), 7.59–7.69 (m, 3H, ArH), 7.74 (br s, 2H, NH_2), 10.56 (s, 1H, OH); ^{13}C-NMR (DMSO-d_6, 150.9 MHz): δ (ppm) = 116.2, 117.3, 126.1, 127.3, 131.0, 131.2, 131.2 (CH); 92.1, 99.8, 113.4, 115.5, 122.2, 132.1, 133.8, 143.2, 151.7, 151.9, 159.8, 160.5, 166.8, 186.2 (C); $C_{21}H_{12}Cl_2N_4O_2S$ (454.00); MS (EI): m/z (%) = 454.0 $[M]^{+•}$ (100), 419.0 $[M^{+•}–Cl]$ (46); HRMS (EI): m/z $[M]^{+•}$ calcd 452.99743, found 452.99754; isocrat. HPLC: 95.0% at 254 nm and 96.8% at 280 nm, t_{ms} = 3.37 min, t_m = 1.12 min (ACN/H_2O 60:40); $λ_{max}$ (nm): 326, 296; gradient HPLC: 96.1% at 254 nm, t_{ms} = 12.1 min, t_m = 1.22 min (0–2 min: ACN/H_2O 10:90, 2–12 min: ACN/H_2O 10:90 → 90:10, 12–20 min: ACN/H_2O 90:10).

<u>3,6-Diamino-2-(3-chlorobenzoyl)-4-(2-chloro-4-methoxyphenyl)thieno[2,3-*b*]pyridine-5-carbonitrile (**3a**)</u>

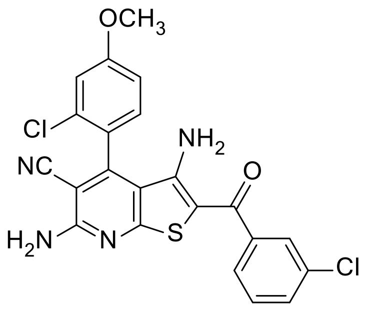

Prepared following GP 4 from 6-amino-4-(2-chlor-4-methoxyphenyl)-2-thioxo-1,2-dihydropyridine-3,5-dicarbonitrile (233 mg, 0.738 mmol), potassium hydroxide solution (10%) (twice 420 µL, 0.750 mmol) and 2-bromo-3'-chloracetophenone (**9**, 175 mg, 0.751 mmol); reaction time: 90 min. Crystallization from ethanol (70% v/v) yielded 346 mg (57%) yellow powder.

Mp: 245–248 °C; IR (KBr): 3477 cm^{-1} and 3342 cm^{-1} (NH), 2215 cm^{-1} (C≡N), 1612 cm^{-1} (C=O); ^{1}H-NMR (DMSO-d_6, 600.1 MHz): δ (ppm) = 3.91 (s, 3H, CH_3), 6.80 (br s, 2H, NH_2), 7.21 (dd, 1H, *J* = 8.6/2.5 Hz, ArH), 7.38 (d, 1H, *J* = 2.5 Hz, ArH), 7.51–7.58 (m, 2H, ArH), 7.61–7.63 (m, 1H, ArH), 7.64–7.68 (m, 2H, ArH), 7.67 (br s, 2H, NH_2); ^{13}C-NMR (DMSO-d_6, 150.9 MHz): δ (ppm) = 55.8 (OCH_3); 114.6, 115.4, 125.5, 126.8, 130.5, 130.6, 130.7 (CH); 91.4, 99.3, 112.7, 114.8, 123.3, 131.9, 133.2, 142.6, 150.8, 151.4, 159.2, 161.3, 166.2, 185.7 (C); $C_{22}H_{14}Cl_2N_4O_2S$ (468.02); calcd C 56.30, H 3.01, N 11.94; found C 56.00, H 2.92, N 11.63; MS (EI): m/z (%) = 468.0 $[M]^{+\bullet}$ (100), 433.0 $[M^{+\bullet}$–Cl] (59); isocrat. HPLC: 99.3% at 254 nm and 98.4% at 280 nm, t_{ms} = 6.22 min, t_m = 1.1 min (ACN/H_2O 60:40); λ_{max} (nm): 327, 295; gradient HPLC: 99.2% at 254 nm, t_{ms} = 13.3 min, t_m = 1.25 min (0–2 min: ACN/H_2O 10:90, 2–12 min: ACN/H_2O 10:90 → 90:10, 12–20 min: ACN/H_2O 90:10).

3,6-Diamino-2-(3-chlorobenzoyl)-4-(2-chloro-4-ethoxyphenyl)thieno[2,3-*b*]pyridine-5-carbonitrile (**3b**)

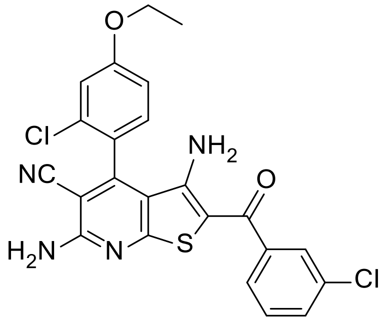

Prepared following GP 4 from 6-amino-4-(2-chloro-4-ethoxyphenyl)-2-thioxo-1,2-dihydropyridine-3,5-dicarbonitrile (177 mg, 0.528 mmol), potassium hydroxide solution (10%) (twice 297 µL, 0.530 mmol) and 2-bromo-3'-chloroacetophenone (**9**, 132 mg, 0.565 mmol); reaction time 70 min. Crystallization from ethanol (70% v/v) yielded 110 mg (43%) yellow powder.

Mp: 253–257 °C; IR (KBr): 3492 cm^{-1}, 3470 cm^{-1} and 3388 cm^{-1} (NH), 2214 cm^{-1} (C≡N), 1604 cm^{-1} (C=O); ^{1}H-NMR (DMSO-d_6, 600.1 MHz): δ (ppm) = 1.39 (t, 3H, *J* = 7.0 Hz, CH_3), 4.18 (q, 2H, *J* = 7.0 Hz, CH_2), 6.61 (br s, 2H, NH_2), 7.18 (dd, 1H, *J* = 8.6/2.5 Hz, ArH), 7.36 (d, 1H, *J* = 2.5 Hz, ArH), 7.49–7.58 (m, 2H, ArH), 7.59–7.68 (m, 3H, ArH), 7.76 (br s, 2H, NH_2); ^{13}C-NMR (DMSO-d_6, 150.9 MHz): δ (ppm) = 14.4 (CH_3); 64.0 (CH_2); 114.9, 115.8, 125.5, 126.8, 130.5, 130.6, 130.6 (CH); 91.4, 99.3, 112.8, 114.9, 123.2, 131.9, 133.3, 142.7, 150.9, 151.4, 159.3, 160.6, 166.3, 185.7 (C); $C_{23}H_{16}Cl_2N_4O_2S$ (482.04); MS (EI): m/z (%) = 482.0 $[M]^{+\bullet}$ (100), 447.0 $[M^{+\bullet}$–Cl] (67); HRMS (EI): m/z $[M]^{+\bullet}$ calcd 482.03655, found 482.03621; isocrat. HPLC: 98.1% at 254 nm and 99.0% at 280 nm, t_{ms} = 8.94 min, t_m = 1.2 min (ACN/H_2O 60:40); λ_{max} (nm): 327, 295; gradient HPLC: 97.0% at 254 nm, t_{ms} = 13.0 min, t_m = 1.25 min (0–2 min: ACN/H_2O 10:90, 2–12 min: ACN/H_2O 10:90 → 90:10, 12–20 min: ACN/H_2O 90:10).

3,6-Diamino-4-[4-(benzyloxy)-2-chlorophenyl]-2-(3-chlorobenzoyl)thieno[2,3-*b*]pyridine-5-carbonitrile (**3c**)

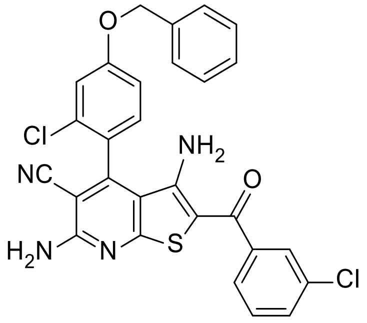

Prepared following GP 4 from 6-amino-4-[4-(benzyloxy)-2-chlorophenyl]-2-thioxo-1,2-dihydropyridine-3,5-dicarbonitrile (130 mg, 0.331 mmol), potassium hydroxide solution (10%) (twice 185 µL, 0.330 mmol) and 2-bromo-3'-chloroacetophenone (**9**, 86 mg, 0.368 mmol); reaction time: 60 min. Crystallization from ethanol (70% v/v) yielded 90 mg (46%) yellow powder.

Mp: 237–241 °C; IR (KBr): 3476 cm^{-1} and 3288 cm^{-1} (NH), 2216 cm^{-1} (C≡N), 1626 cm^{-1} (C=O); ^{1}H-NMR (DMSO-d_6, 600.1 MHz): δ (ppm) = 5.24 (s, 2H, CH_2), 6.72 (br s, 2H, NH_2), 7.29 (dd, 1H, J = 8.6/2.5 Hz, ArH), 7.36–7.43 (m, 1H, ArH), 7.43–7.48 (m, 2H, ArH), 7.49 (d, 1H, J = 2.5 Hz, ArH), 7.51–7.60 (m, 4H, ArH), 7.60–7.64 (m, 1H, ArH), 7.64–7.69 (m, 2H, ArH) 7.77 (br s, 2H, NH_2); ^{13}C-NMR (DMSO-d_6, 150.9 MHz): δ (ppm) = 70.1 (CH_2); 115.2, 116.2, 125.5, 126.8, 128.1 (2 C), 128.2, 128.5 (2 C), 130.5, 130.6, 130.7 (CH); 91.4, 99.4, 112.8, 114.9, 123.6, 132.0, 133.3, 136.1, 142.6, 150.8, 151.4, 159.3, 160.5, 166.3, 185.7 (C); $C_{28}H_{18}Cl_2N_4O_2S$ (545.44); MS (EI): m/z (%) = 544.0 $[M]^{+\bullet}$ (100); HRMS (EI): m/z $[M]^{+\bullet}$ calcd 544.0522, found 544.0517; isocrat. HPLC: 96.3% at 254 nm and 97.4% at 280 nm, t_{ms} = 5.69 min, t_m = 1.2 min (ACN/H_2O 60:40); λ_{max} (nm): 327, 294; gradient HPLC: 95.9% at 254 nm, t_{ms} = 14.6 min, t_m = 1.25 min (0–2 min: ACN/H_2O 10:90, 2-12 min: ACN/H_2O 10:90 → 90:10, 12–20 min: ACN/H_2O 90:10).

3,6-Diamino-2-(3-chlorobenzoyl)-4-[2-chloro-4-(2-hydroxyethoxy)phenyl]thieno[2,3-*b*]-pyridine-5-carbonitrile (**3d**)

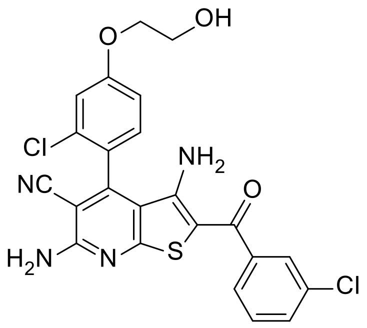

Prepared following GP 4 from 6-amino-4-[2-chloro-4-(2-hydroxyethoxy)phenyl]-2-thioxo-1,2-dihydropyridine-3,5-dicarbonitrile (662 mg, 1.90 mmol), potassium hydroxide solution (10%) (four times 250 µL, 1.80 mmol) and 2-bromo-3'-chloroacetophenone (**9**, 395 mg, 1.69 mmol); reaction time 5.5 h. Crystallization from ethanol (70% v/v) yielded a yellow powder (428 mg, 57%).

Mp: 145–147 °C; IR (KBr): 3461 cm^{-1} and 3302 cm^{-1} (NH), 2213 cm^{-1} (C≡N), 1604 cm^{-1} (C=O); ^{1}H-NMR (DMSO-d_6, 600.1 MHz): δ (ppm) = 3.75–3.79 (m, 2H, CH_2), 4.13–4.17 (m, 2H, CH_2), 4.99 (t, *J* = 5.4 Hz, 1H, OH), 6.79 (br s, 2H, NH_2), 7.22 (dd, *J* = 8.6/2.4 Hz, 1H, ArH), 7.39 (d, *J* = 2.4 Hz, 1H, ArH), 7.52–7.71 (m, 5H, ArH), 7.76 (br s, 2H, NH_2); ^{13}C-NMR (DMSO-d_6, 150.9 MHz): δ (ppm) = 59.3, 70.3 (CH_2); 115.0, 115.9, 125.5, 126.8, 130.5, 130.6, 130.6 (CH); 91.5, 99.5, 112.9, 123.4, 132.0, 133.3, 142.7, 150.9, 151.5, 155.1, 159.3, 160.9, 166.3, 185.8 (C); $C_{23}H_{16}Cl_2N_4O_3S$ (498.03); calcd C 55.32, H 3.23, N 11.22; found C 54.91, H 3.10, N 10.86; MS (EI): m/z (%) = 498.0 $[M]^{+\bullet}$ (100), 463.0 $[M^{+\bullet}$–Cl] (58); isocrat. HPLC: 98.8% at 254 nm and 98.3% at 280 nm, t_{ms} = 6.16 min, t_m = 1.2 min (ACN/H_2O 50:50); λ_{max} (nm): 328, 296; gradient HPLC: 96.6% at 254 nm, t_{ms} = 11.8 min, t_m = 1.25 min (0–2 min: ACN/H_2O 10:90, 2–12 min: ACN/H_2O 10:90 → 90:10, 12–20 min: ACN/H_2O 90:10).

<u>3,6-Diamino-2-(3-chlorobenzoyl)-4-{2-chloro-4-[(2,2-dimethyl-1,3-dioxolan-4-yl)methoxy]phenyl}thieno[2,3-*b*]pyridine-5-carbonitrile (**3e**)</u>

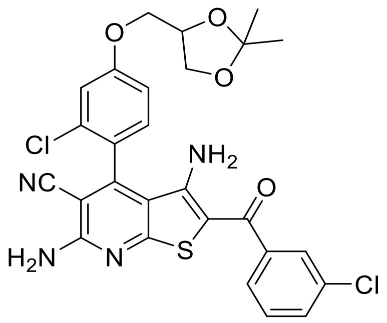

Prepared following GP 4 from 6-amino-4-{2-chloro-4-[(2,2-dimethyl-1,3-dioxolan-4-yl)methoxy]phenyl}-2-thioxo-1,2-dihydropyridine-3,5-dicarbonitrile (489 mg, 1.17 mmol), potassium hydroxide solution (10%) (twice 655 µL, 1.17 mmol) and 2-bromo-3’-chloroacetophenone (**9**, 274 mg, 1.17 mmol); reaction time 2 h. The precipitate was filtered off with suction and was dissolved in acetone. After addition of silica gel (3 g) the slurry was evaporated to dryness. The product was eluted by column chromatography (petrol ether/ethyl acetate 1:1). The resulting solid was further crystallized from petrol ether/ethyl acetate (3:1) to yield a yellow powder (370 mg, 55%).

Mp: 191–193 °C (dec.); IR (KBr): 3468 cm^{-1}, 3410 cm^{-1} and 3316 cm^{-1} (NH), 2219 cm^{-1} (C≡N), 1605 cm^{-1} (C=O); ^{1}H-NMR (DMSO-d_6, 600.1 MHz): δ (ppm) = 1.33 (s, 3H, CH_3), 1.39 (s, 3H, CH_3), 3.80–3.82 (m, 1H, CH_2), 4.11–4.16 (m, 2H, CH_2), 4.18–4.22 (m, 1H, CH), 4.44–4.47 (m, 1H, CH_2), 7.23 (dd, *J* = 8.6/2.6 Hz, 1H, ArH), 7.42 (d, *J* = 2.5 Hz, 1H, ArH), 7.53–7.57 (m, 2H, ArH), 7.62 (d, *J* = 8.5 Hz, 1H, ArH), 7.64–7.68 (m, 2H, ArH), 7.76 (br s, 2H, NH_2; the second NH_2-signal was not detectable); ^{13}C-NMR (DMSO-d_6, 150.9 MHz): δ (ppm) = 25.4, 26.6 (CH_3); 65.5, 69.4/69.4 (double signal) (CH_2); 73.5, 115.1/115.2 (double signal), 115.9/116.0 (double signal), 125.6, 126.9, 130.5, 130.7, 130.8 (CH); 91.5, 99.5, 109.0, 112.8, 114.9, 123.7, 132.0/132.0 (double signal), 133.3, 142.7, 150.9, 151.5, 159.4, 160.5, 166.4, 185.8 (C); $C_{27}H_{22}Cl_2N_4O_4S$ (569.46); calcd C 56.95, H 3.89, N 9.84; found C 56.96, H 3.84, N 9.68; MS (EI): m/z (%) = 568.0 $[M]^{+\bullet}$ (100), 533.0 $[M\text{-}Cl]^{+\bullet}$ (32); isocrat. HPLC: 98.8% at 254 nm and 97.2% at 280 nm, t_{ms} = 5.11 min, t_m = 1.12 min (ACN/H_2O 50:50); λ_{max} (nm): 328, 290; gradient HPLC: 98.5% at 254 nm, t_{ms} = 13.76 min, t_m = 1.22 min (0–2 min: ACN/H_2O 10:90, 2–12 min: ACN/H_2O 10:90 → 90:10, 12–20 min: ACN/H_2O 90:10).

3,6-Diamino-2-(3-chlorobenzoyl)-4-[2-chloro-4-(2,3-dihydroxypropoxy)phenyl]thieno-[2,3-*b*]pyridine-5-carbonitrile (**3f**)

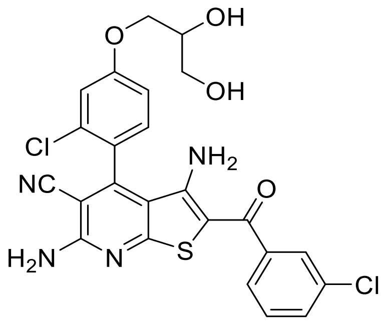

3,6-Diamino-4-{2-chloro-4-[(2,2-dimethyl-1,3-dioxolan-4-yl)methoxy]-phenyl}-2-(3-chlorobenzoyl)thieno[2,3-*b*]pyridine-5-carbonitrile (**3e**, 232 mg, 0.407 mmol) was dissolved in dichloromethane (3 mL). A mixture of trifluoroacetic acid and water 5:1 (3.7 mL) was added. The mixture was stirred for 3 h at room temperature. Upon addition of water (17 mL) a yellow solid precipitated, which was filtered off with suction. Crystallization from petrol ether/ethyl acetate (3:1) yielded a yellow powder (160 mg, 74%).

Mp: 221–225 °C (dec.); IR (KBr): 3466 cm^{-1}, 3314 cm^{-1} and 3163 cm^{-1} (NH), 2216 cm^{-1} (C≡N), 1605 cm^{-1} (C =O); ^{1}H-NMR (DMSO-d_6, 600.1 MHz): δ (ppm) = 3.44–3.56 (m, 2H, CH_2), 3.81–3.91 (m, 1H, CH), 3.99–4.07 (m, 1H, CH_2), 4.11–4.19 (m, 1H, CH_2), 4.70–4.81 (m, 1H, OH), 5.03–5.13 (m, 1H, OH), 7.20 (dd, J = 8.6/2.6 Hz, 1H, ArH), 7.37 (d, J = 2.5 Hz, 1H, ArH), 7.52–7.57 (m, 2H, ArH), 7.62 (d, J = 8.5 Hz, 1H, ArH), 7.64–7.68 (m, 2H, ArH), 7.76 (br s, 2H, NH_2; the second NH_2-signal was not detectable); ^{13}C-NMR (DMSO-d_6, 150.9 MHz): δ (ppm) = 62.5, 70.4 (CH_2); 69.8/69.8 (double signal), 115.0/115.1 (double signal), 116.0/116.0 (double signal), 125.6, 126.9, 130.5, 130.7, 130.7 (CH); 91.5, 99.4, 112.9, 115.0, 123.4, 132.0, 133.3, 142.7, 151.0, 151.5, 159.4, 160.9, 166.4, 185.8 (C); $C_{24}H_{18}Cl_2N_4O_4S$ (529.39); calcd C 54.45, H 3.43, N 10.58; found C 54.47, H 3.36, N 10.39; MS (EI): m/z (%) = 528.0 $[M]^{+\bullet}$ (100), 493.0 $[M^{+\bullet}$-Cl] (52); isocrat. HPLC: 96.9% at 254 nm and 96.8% at 280 nm, t_{ms} = 4.31 min, t_m = 1.12 min (ACN/H_2O 30:70); λ_{max} (nm): 331, 299; gradient HPLC: 95.4% at 254 nm, t_{ms} = 10.9 min, t_m = 1.22 min (0–2 min: ACN/H_2O 10:90, 2–12 min: ACN/H_2O 10:90 → 90:10, 12–20 min: ACN/H_2O 90:10).

tert-Butyl 2-{3-chloro-4-[3,6-diamino-2-(3-chlorobenzoyl)-5-cyanothieno[2,3-*b*]pyridine4-yl]phenoxy}acetate

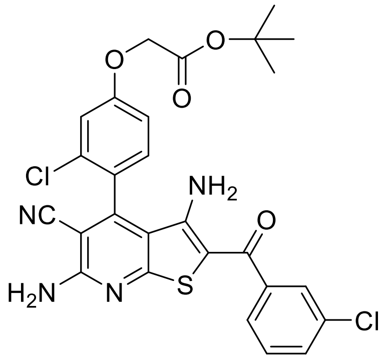

Prepared following GP 4 from *tert*-Butyl 2-[4-(6-amino-3,5-dicyano-2-thioxo-1,2-dihydropyridin-4-yl)-3-chlorophenoxy]acetate (528 mg, 1.26 mmol), potassium hydroxide solution (10%) (twice 710 µL, 1.27 mmol) and 2-bromo-3'-chloroacetophenone (**9**, 294 mg, 1.26 mmol); reaction time 2.0 h. After dissolving the mixture in acetone (5 mL), silica gel (3 g) is added and the slurry is evaporated to dryness. Column chromatography (petrol ether/ethyl acetate 2:1) yielded 69 mg (8%) yellow powder, which was used for the subsequent procedure without further purification.

Mp: 192–194 °C; IR (KBr): 3475 cm^{-1} and 3354 cm^{-1} (NH), 2217 cm^{-1} (C≡N), 1607 cm^{-1} (C=O); $C_{27}H_{22}Cl_2N_4O_4S$ (568.07); MS (EI): m/z (%) = 57.1 $[M^{+\bullet}$-511] (100), 568.1 $[M]^{+\bullet}$ (50); gradient HPLC: 96.0% at 254 nm, t_{ms} = 14.2 min, t_m = 1.22 min (0–2 min: ACN/H_2O 10:90, 2–12 min: ACN/H_2O 10:90 → 90:10, 12–20 min: ACN/H_2O 90:10).

2-{3-Chloro-4-[3,6-diamino-2-(3-chlorobenzoyl)-5-cyanothieno[2,3-*b*]pyridin-4-yl]phenoxy}acetic acid (**3g**)

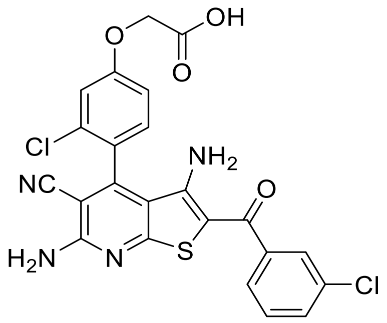

Preparation following GP 5 from *tert*-butyl 2-{3-chloro-4-[3,6-diamino-2-(3-chlorobenzoyl)-5-cyanothieno[2,3-*b*]pyridin-4-yl]phenoxy}acetate (60.0 mg, 0.105 mmol), in dry dichloromethane (2.5 mL) and trifluoroacetic acid (1.3 mL); reaction time 14 h, yielded a yellow powder (26 mg, 48%).

Mp: 219–221 °C; IR (KBr): 3477 cm^{-1} and 3327 cm^{-1} (NH), 2224 cm^{-1} (C≡N), 1636 cm^{-1} (C=O); ^{1}H-NMR (DMSO-d_6, 600.1 MHz): δ (ppm) = 4.86 (s, 2H, CH_2), 6.78 (br s, 2H, NH_2), 7.21 (dd, J = 8.6/2.5 Hz, 1H, ArH), 7.40 (d, J = 2.5 Hz, 1H, ArH), 7.53–7.58 (m, 2H, ArH), 7.62 (d, J = 8.4 Hz, 1H, ArH), 7.64–7.67 (m, 2H, ArH), 7.72–7.83 (m, 2H, ArH), 13.19 (s, 1H, COOH); ^{13}C-NMR (DMSO-d_6, 150.9 MHz): δ (ppm) = 65.1 (CH_2); 115.1, 116.3, 125.6, 126.9, 130.6, 130.7, 130.8 (CH); 91.4, 99.5, 112.8, 114.9, 124.0, 132.0, 133.3, 142.7, 150.8, 151.4, 159.4, 159.9, 166.3, 169.6, 185.8 (C); $C_{23}H_{14}Cl_2N_4O_4S$ (513.35); MS (EI): m/z (%) = 512.0 $[M]^{+\bullet}$ (100), 477.0 $[M^{+\bullet}$-Cl] (42); HRMS (EI): m/z $[M]^{+\bullet}$ calcd 512.01073, found 512.00997; isocrat. HPLC: 99.6% at 254 nm and 99.9% at 280 nm, t_{ms} = 3.96 min, t_m = 1.12 min (ACN/buffer 50:50); λ_{max} (nm): 328, 296; gradient HPLC: 98.4% at 254 nm, t_{ms} = 9.27 min, t_m = 1.22 min (0–2 min: ACN/H_2O 10:90, 2–12 min: ACN/H_2O 10:90 → 90:10, 12–20 min: ACN/H_2O 90:10).

<u>3,6-Diamino-2-(3-chlorobenzoyl)-4-[2-chloro-4-(dimethylamino)phenyl]thieno[2,3-*b*]-pyridine-5-carbonitrile (**4a**)</u>

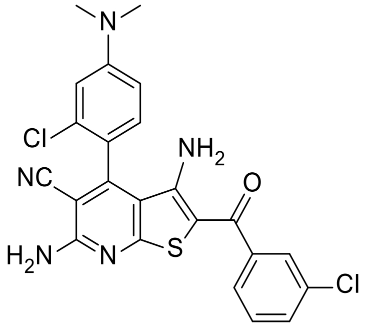

Prepared following GP 4 from 6-amino-4-[2-chloro-4-(dimethylamino)phenyl]-2-thioxo-1,2-dihydropyridine-3,5-dicarbonitrile (188 mg, 0.446 mmol), potassium hydroxide solution (10%) (twice 250 µL, 0.892 mmol) and 2-bromo-3'-chloroacetophenone (**9**, 104 mg, 0.445 mmol); reaction time 6 h. Crystallization from ethanol (70% v/v) yielded a yellow powder (119 mg, 25%).

Mp: 312–313 °C (dec.); IR (KBr): 3466 cm^{-1} and 3352 cm^{-1} (NH), 2216 cm^{-1} (C≡N), 1610 cm^{-1} (C=O); ^{1}H-NMR (DMSO-d_6, 600.1 MHz): δ (ppm) = 3.05 (s, 6H, CH_3), 6.80 (br s, 2H, NH_2), 6.89 (dd, J = 8.8/2.5 Hz, 1H, ArH), 6.97 (d, J = 2.5 Hz, 1H, ArH), 7.35 (d, J = 8.7 Hz, 1H, ArH), 7.51–7.58 (m, 1H, ArH), 7.59–7.78 (m, 5H, ArH, NH_2 signal is superposed by ArH signals); ^{13}C-NMR (DMSO-d_6, 150.9 MHz): δ (ppm) = 39.7 (2 CH_3); 111.2, 111.9, 125.6, 126.8, 130.0, 130.5, 130.6 (CH); 91.9, 99.3, 113.2, 115.1, 117.1, 131.9, 133.3, 142.8, 151.6, 151.7, 152.1, 159.4, 166.2, 185.6 (C); $C_{23}H_{17}Cl_2N_5OS$ (481.05); calcd C 57.27, H 3.55, N 14.52; found C 57.11, H 3.46, N 13.99; MS (EI): m/z (%) = 481.0 $[M]^{+•}$ (45), 224.2 [$M^{+•}$-257] (100); HRMS (ESI): m/z $[M+Na]^+$ calcd 504.04231, found 504.04219; isocrat. HPLC: 97.1% at 254 nm and 97.9% at 280 nm, t_{ms} = 3.69 min, t_m = 1.2 min (ACN/H_2O 70:30); λ_{max} (nm): 325, 266; gradient HPLC: 97.6% at 254 nm, t_{ms} = 13.9 min, t_m = 1.25 min (0–2 min: ACN/H_2O 10:90, 2–12 min: ACN/H_2O 10:90 → 90:10, 12–20 min: ACN/H_2O 90:10).

3,6-Diamino-2-(3-chlorobenzoyl)-4-[2-chloro-4-(diethylamino)phenyl]thieno[2,3-*b*]-pyridine-5-carbonitrile (**(+)-4b** and **(-)-4b**)

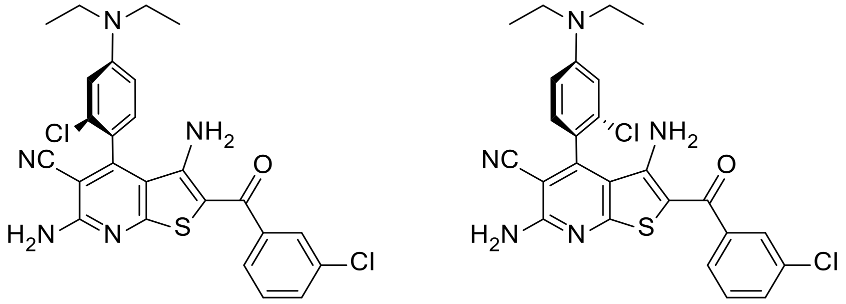

Prepared following GP 4 from 6-amino-4-[2-chloro-4-(diethylamino)phenyl]-2-thioxo-1,2-dihydropyridine-3,5-dicarbonitrile (554 mg, 1.55 mmol), potassium hydroxide solution (10%) (four times 250 µL, 1.80 mmol) and 2-bromo-3'-chloroacetophenone (**9**, 362 mg, 1.55 mmol); reaction time 130 min. Crystallization from ethanol (70% v/v) yielded a yellow powder (197 mg, 27%).

Mp: 285–287 °C (dec.); IR (KBr): 3468 cm^{-1} and 3367 cm^{-1} (NH), 2215 cm^{-1} (C≡N), 1610 cm^{-1} (C=O); ^{1}H-NMR (DMSO-d_6, 600.1 MHz): δ (ppm) = 1.66 (t, J = 7.0 Hz, 6H, 2 CH_3), 3.44 (q, J = 7.0 Hz, 4H, 2 CH_2), 6.80 (br s, 2H, NH_2), 6.85 (dd, J = 8.8/2.6 Hz, 1H, ArH), 6.90 (d, J = 2.5 Hz, 1H, ArH), 7.30 (d, J = 8.7 Hz, 1H, ArH), 7.53–7.57 (m, 1H, ArH), 7.60–7.64 (m, 1H, ArH), 7.65–7.67 (m, 2H, ArH), 7.70 (br s, 2H, NH_2, superimosed by ArH signals at 7.65); ^{13}C-NMR (DMSO-d_6, 150.9 MHz): δ (ppm) = 12.0 (2 CH_3); 43.4 (2 CH_2); 110.4, 110.9, 125.4, 126.6, 130.1, 130.3, 130.4 (CH); 91.9, 99.2, 113.3, 115.3, 116.2, 132.1, 133.3, 142.8, 149.7, 151.7, 151.9, 159.5, 166.3, 185.6 (C); $C_{25}H_{21}Cl_2N_5OS$ (509.1); calcd C 58.83, H 4.15, N 13.72; found C 58.51, H 4.18, N 13.33; MS (EI): m/z (%) = 509.1 $[M]^{+\bullet}$ (100), 474.1 $[M^{+\bullet}$-Cl] (89); isocrat. HPLC: 98.9% at 254 nm and 99.4% at 280 nm, t_{ms} = 6.87 min, t_m = 1.12 min (ACN/H_2O 70:30); λ_{max} (nm): 326, 271, 294; gradient HPLC: 97.6% at 254 nm, t_{ms} = 14.6 min, t_m = 1.25 min (0–2 min: ACN/H_2O 10:90, 2–12 min: ACN/H_2O 10:90 → 90:10, 12–20 min: ACN/H_2O 90:10); $[\alpha]_D^{20}$: **34b₁** +41.0 (c = 0.004 g/cm^3 in DCM), **34b₂** –42.7 (c = 0.004 g/cm^3 in DCM).

3,6-Diamino-2-(3-chlorobenzoyl)-4-[2-chloro-4-(pyrrolidin-1-yl)phenyl]thieno[2,3-*b*]-pyridine-5-carbonitrile (**4c**)

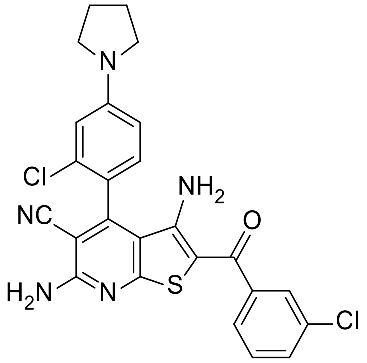

Prepared following GP 4 from 6-amino-4-[2-chloro-4-(pyrrolidin-1-yl)phenyl]-2-thioxo-1,2-dihydropyridine-3,5-dicarbonitrile (600 mg, 1.70 mmol), potassium hydroxide solution (10%) (four times 250 µL, 1.80 mmol) and 2-bromo-3'-chloroacetophenone (**9**, 394 mg, 1.70 mmol); reaction time 4 h. After dissolving the mixture in acetone (5 mL), silica gel (3 g) was added and the slurry was evaporated to dryness. Column chromatography (toluene/ethyl acetate 1:1) yielded yellow powder (131 mg, 15%).

Mp: 262–265 °C; IR (KBr): 3462 cm^{-1} and 3207 cm^{-1} (NH), 2217 cm^{1} (C≡N), 1607 cm^{-1} (C=O); ^{1}H-NMR ($CDCl_3$, 600.1 MHz): δ (ppm) = 1.97–2.21 (m, 4H, 2 CH_2), 3.22–3.48 (m, 4H, 2 CH_2), 5.57 (br s, 2H, NH_2), 6.60 (dd, *J* = 8.5/2.4 Hz, 1H, ArH), 6.72 (d, *J* = 2.4 Hz, 1H, ArH), 7.15 (d, *J* = 8.5 Hz, 1H, ArH), 7.40–7.74 (m, 4H, ArH), the second NH_2-signal was not detectable; ^{13}C-NMR ($CDCl_3$, 150.9 MHz): δ (ppm) = 25.3 (2 CH_2); 47.4 (2 CH_2); 110.6, 112.2, 125.4, 127.6, 129.5, 129.6, 130.7 (CH); 92.5, 101.7, 115.3, 115.4, 116.2, 132.8, 134.3, 142.3, 149.6, 151.6, 151.6, 158.3, 166.5, 187.4 (C); $C_{25}H_{19}Cl_2N_5OS$ (507.07); calcd C 59.06, H 3.77, N 13.78; found C 58.96, H 3.65, N 13.36; MS (EI): m/z (%) = 507.1 $[M]^{+•}$ (87), 472.1 $[M^{+•}$-Cl] (100); isocrat. HPLC: 97.0% at 254 nm and 98.5% at 280 nm, t_{ms} = 6.15 min, t_m = 1.12 min (ACN/H_2O 70:30); $λ_{max}$ (nm): 326, 270, 296; gradient HPLC: 98.7% at 254 nm, t_{ms} = 14.9 min, t_m = 1.25 min (0–2 min: ACN/H_2O 10:90, 2–12 min: ACN/H_2O 10:90 → 90:10, 12–20 min: ACN/H_2O 90:10).

3,6-Diamino-4-[2-chloro-4-(piperidin-1-yl)phenyl]-2-(3-chlorobenzoyl)thieno[2,3-*b*]-pyridine-5-carbonitrile (**4d**)

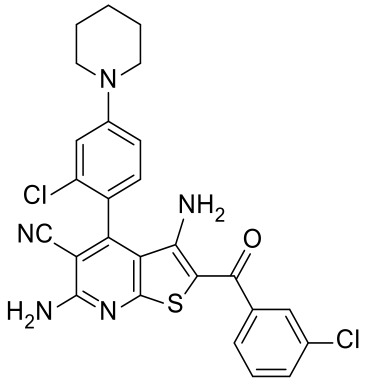

Prepared following GP 4 from 6-amino-4-[2-chloro-4-(piperidin-1-yl)phenyl]-2-thioxo-1,2-dihydropyridine-3,5-dicarbonitrile (372 mg, 1.01 mmol), potassium hydroxide solution (10%) (four times 250 µL, 1.80 mmol) and 2-bromo-3'-chloroacetophenone (**9**, 235 mg, 1.00 mmol); reaction time 5 h. After dissolving the mixture in acetone (5 mL), silica gel (3 g) was added and the slurry was evaporated to dryness. Column chromatography (toluene/ethyl acetate 1:1) yielded yellow powder (112 mg, 21%).

Mp: 220–223 °C; IR (KBr): 3468 cm^{-1} and 3293 cm^{-1} (NH), 2218 cm^{-1} (C≡N), 1604 cm^{-1} (C=O); ^{1}H-NMR ($CDCl_3$, 600.1 MHz): δ (ppm) = 1.64–1.69 (m, 2H, CH_2), 1.70–1.76 (m, 4H, 2 CH_2), 3.26–3.41 (m, 4H, 2 CH_2), 5.60 (br s, 2H, NH_2), 6.95 (dd, *J* = 8.7/2.5 Hz, 1H, ArH), 7.06 (d, *J* = 2.5 Hz, 1H, ArH), 7.18 (d, *J* = 8.6 Hz, 1H, ArH), 7.40 (d, *J* = 7.8 Hz, 1H, ArH), 7.47 (dd, *J* = 8.0/2.1 Hz, 1H, ArH), 7.63–7.67 (m, 1H, ArH), 7.74 (d, *J* = 1.9 Hz, 1H, ArH), the second NH_2-signal was not detectable; ^{13}C-NMR ($CDCl_3$, 150.9 MHz): δ (ppm) = 23.9 (CH_2); 25.2 (2 CH_2), 48.5 (2 CH_2); 113.6, 115.2, 125.4, 127.6, 129.5, 129.7, 130.8 (CH); 92.2, 101.8, 115.1, 115.4, 118.9, 132.9, 134.4, 142.2, 151.1, 151.4, 153.4, 158.2, 166.5, 187.4 (C); $C_{26}H_{21}Cl_2N_5OS$ (521.08); calcd C 59.77, H 4.05, N 13.41; found C 59.88, H 3.97, N 13.11; MS (EI): m/z (%) = 521.1 $[M]^{+\bullet}$ (85), 486.1 $[M^{+\bullet}$-Cl] (100); isocrat. HPLC: 97.3% at 254 nm and 98.1% at 280 nm, t_{ms} = 7.01 min, t_m = 1.12 min (ACN/H_2O 45:55); λ_{max} (nm): 325, 270, 293; gradient HPLC: 96.5% at 254 nm, t_{ms} = 15.3 min, t_m = 1.25 min (0–2 min: ACN/H_2O 10:90, 2–12 min: ACN/H_2O 10:90 → 90:10, 12–20 min: ACN/H_2O 90:10).

3,6-Diamino-2-(3-chlorobenzoyl)-4-(2-chloro-4-morpholinophenyl)thieno[2,3-*b*]pyridine-5-carbonitrile (**4e**)

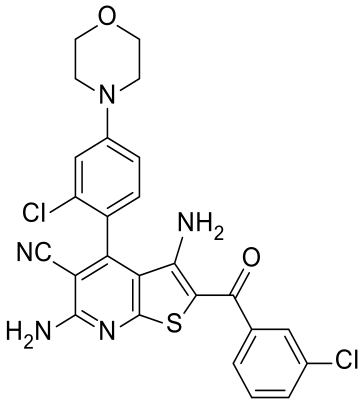

Prepared following GP 4 from 6-amino-4-(2-chloro-4-morpholinophenyl)-2-thioxo-1,2-dihydropyridine-3,5-dicarbonitrile (386 mg, 1.04 mmol), potassium hydroxide solution (10%) (twice 280 µL, 1.00 mmol) and 2-bromo-3'-chloroacetophenone (**9**, 250 mg, 1.04 mmol); reaction time 2.5 h. Crystallization from ethanol (70% v/v) yielded a yellow powder (137 mg, 26%).

Mp: 262–271 °C (dec.); IR (KBr): 3470 cm^{-1}, 3301 cm^{-1} and 3208 cm^{-1} (NH), 2219 cm^{-1} (C≡N), 1623 cm^{-1} (C=O); ^{1}H-NMR (DMSO-d_6, 600.1 MHz): δ (ppm) = 3.31–3.35 (m, 4H, 2 CH_2), 3.73–3.82 (m, 4H, 2 CH_2), 6.72 (br s, 2H, NH_2), 7.16 (dd, *J* = 8.6/2.5 Hz, 1H, ArH), 7.27 (d, *J* = 2.5 Hz, 1H, ArH), 7.42 (d, *J* = 8.6 Hz, 1H, ArH), 7.50–7.59 (m, 1H, ArH), 7.61–7.68 (m, 3H, ArH), 7.73 (br s, 2H, NH_2); ^{13}C-NMR (DMSO-d_6, 150.9 MHz): δ (ppm) = 46.7 (2 CH_2), 65.6 (2 CH_2); 113.3, 114.4, 125.4, 126.6, 130.0, 130.3, 130.4 (CH); 91.7, 99.3, 113.6, 115.1, 120.2, 132.0, 133.3, 142.8, 151.4, 151.6, 152.9, 159.4, 166.3, 185.7 (C); $C_{25}H_{19}Cl_2N_5O_2S$ (523.06); MS (EI): m/z (%) = 523.0 $[M]^{+\bullet}$ (100), 488.1 $[M^{+\bullet}–Cl]$ (82); HRMS (EI): m/z $[M]^{+\bullet}$ calcd 523.0631, found 523.0630; isocrat. HPLC: 95.2% at 254 nm and 95.4% at 280 nm, t_{ms} = 3.28 min, t_m = 1.2 min (ACN/H_2O 70:30); λ_{max} (nm): 326, 261, 297; gradient HPLC: 95.2% at 254 nm, t_{ms} = 13.2 min, t_m = 1.25 min (0–2 min: ACN/H_2O 10:90, 2–12 min: ACN/H_2O 10:90 → 90:10, 12–20 min: ACN/H_2O 90:10).

tert-Butyl [2-({3-chloro-4-[3,6-diamino-2-(3-chlorobenzoyl)-5-cyanothieno[2,3-*b*]pyridin-4-yl]phenyl}{methyl}amino)ethyl]carbamate (**4f**)

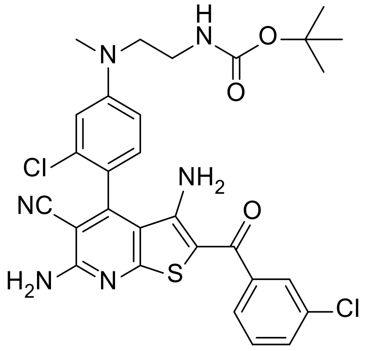

Prepared following GP 4 from *tert*-butyl (2-{[4-(6-amino-3,5-dicyano-2-thioxo-1,2-dihydropyridin-4-yl)-3-chlorophenyl][methyl]amino}ethyl)carbamate (528 mg, 1.15 mmol), potassium hydroxide solution (10%) (twice 650 µL, 1.15 mmol) and 2-bromo-3'-chloroacetophenone (**9**, 270 mg, 1.15 mmol); reaction time 2 h. After dissolving the mixture in acetone (5 mL), silica gel (3 g) was added and the slurry was evaporated to dryness. Column chromatography (petrol ether/ethyl acetate 3:1) yielded a yellow powder (385 mg, 63%).

Mp: 148–150 °C; IR (KBr): 3467 cm^{-1} and 3369 cm^{-1} (NH), 2214 cm^{-1} (C≡N), 1606 cm^{-1} (C=O); ^{1}H-NMR (DMSO-d_6, 600.1 MHz): δ (ppm) = 1.38 (s, 9H, 3 CH_3), 3.01 (s, 3H, CH_3), 3.16 (t, J = 6.7 Hz, 2H, CH_2), 3.47 (t, J = 6.7 Hz, 2H, CH_2), 6.90 (dd, J = 8.8/2.6 Hz, 1H, ArH), 6.95 (d, J = 2.6 Hz, 1H, ArH), 6.98 (s, 1H, NH), 7.33 (d, J = 8.7 Hz, 1H, ArH), 7.46 (br s, 2H, NH_2), 7.53–7.56 (m, 1H, ArH), 7.61–7.67 (m, 3H, ArH), 7.70 (br s, 2H, NH_2); ^{13}C-NMR (DMSO-d_6, 150.9 MHz): δ (ppm) = 29.6 (3 CH_3), 39.5 (CH_3); 38.3, 52.1 (CH_2); 112.3, 113.1, 127.0, 128.3, 131.5, 131.9, 132.1 (CH); 77.8, 91.9, 99.3, 113.3, 115.2, 116.9, 131.9, 133.3, 142.8, 151.2, 151.7, 151.8, 155.8, 159.5, 166.3, 185.7 (C); $C_{29}H_{28}Cl_2N_6O_3S$ (611.54); calcd C 56.96, H 4.62, N 13.74; found C 56.76, H 4.56, N 13.58; MS (EI): m/z (%) = 610.1 $[M]^{+\bullet}$ (13), 480.1 $[M^{+\bullet}-C_6H_{12}NO_2]$ (100); isocrat. HPLC: 99.3% at 254 nm and 99.6% at 280 nm, t_{ms} = 4.69 min, t_m = 1.12 min (ACN/H_2O 70:30); λ_{max} (nm): 326, 268; gradient HPLC: 98.8% at 254 nm, t_{ms} = 14.2 min, t_m = 1.22 min (0–2 min: ACN/H_2O 10:90, 2–12 min: ACN/H_2O 10:90 → 90:10, 12–20 min: ACN/H_2O 90:10).

tert-Butyl 4-{3-chloro-4-[3,6-diamino-2-(3-chlorobenzoyl)-5-cyanothieno[2,3-*b*]pyridin-4-yl]phenyl}piperazine-1-carboxylate (**4g**)

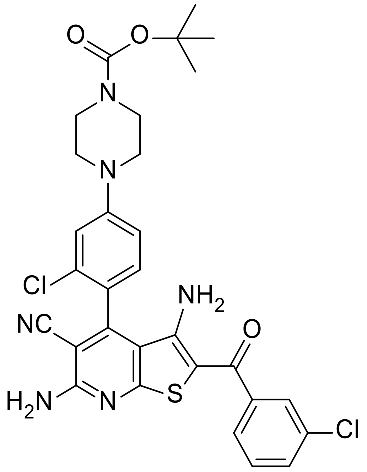

Prepared following GP 4 from *tert*-butyl 4-[4-(6-amino-3,5-dicyano-2-thioxo-1,2-dihydropyridin-4-yl)-3-chlorophenyl]piperazine-1-carboxylate (471 mg, 1.00 mmol), potassium hydroxide solution (10%) (twice 250 µL, 0.892 mmol) and 2-bromo-3'-chloroacetophenone (**9**, 235 mg, 1.01 mmol); reaction time 3.5 h. Crystallization from ethanol (70% v/v) yielded a yellow powder (209 mg, 34%).

Mp: 125–134 °C; IR (KBr): 3468 cm^{-1} and 3212 cm^{-1} (NH), 2216 cm^{-1} (C≡N), 1602 cm^{-1} (C=O); ^{1}H-NMR (DMSO-d_6, 600.1 MHz): δ (ppm) = 1.50 (s, 9H, 3 CH_3), 3.49–3.65 (m, 8H, 4 CH_2), 6.55–7.18 (br s, 2H, NH_2), 7.20 (dd, 1H, *J* = 8.8/2.5 Hz, ArH), 7.32 (d, *J* = 2.4 Hz, 1H, ArH), 7.46 (d, *J* = 8.7 Hz, 1H, ArH), 7.58–7.61 (m, 1H, ArH), 7.65–7.67 (m, 1H, ArH), 7.69–7.73 (m, 2H, ArH), 7.80 (br s, 2H, NH_2); ^{13}C-NMR (DMSO-d_6, 150.9 MHz): δ (ppm) = 28.1 (3 CH_3); 46.6 (4 CH_2); 113.7, 114.8, 125.4, 126.6, 130.0, 130.3, 130.4 (CH); 79.2, 91.7, 99.3, 113.1, 115.1, 120.0, 132.0, 133.3, 142.8, 151.4, 151.6, 152.5, 153.9, 159.4, 166.3, 185.7 (C); $C_{30}H_{28}Cl_2N_6O_3S$ (622.13); MS (EI): m/z (%) = 622.1 $[M]^{+\bullet}$ (21), 522.1 $[M^{+\bullet}–C_5H_9O_2]$ (78), 57.1 $[M^{+\bullet}–565.1]$ (100); HRMS (EI): m/z $[M]^{+\bullet}$ calcd 622.13152, found 622.13148; isocrat. HPLC: 97.8% at 254 nm and 99.5% at 280 nm, t_{ms} = 4.20 min, t_m = 1.2 min (ACN/H_2O 75:25); λ_{max} (nm): 327, 263, 295; gradient HPLC: 98.2% at 254 nm, t_{ms} = 14.6 min, t_m = 1.25 min (0–2 min: ACN/H_2O 10:90, 2–12 min: ACN/H_2O 10:90 → 90:10, 12–20 min: ACN/H_2O 90:10).

<u>3,6-Diamino-2-(3-chlorobenzoyl)-4-(2-chloro-4-{[2-(dimethylamino)ethyl][methyl]-amino}phenyl)thieno[2,3-*b*]pyridine-5-carbonitrile (**4h**)</u>

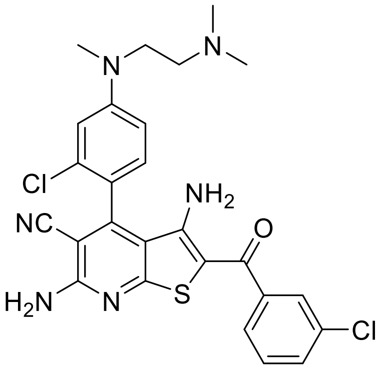

Prepared following GP 4 from 6-amino-4-(2-chloro-4-{[2-(dimethylamino)-ethyl][methyl]amino}phenyl)-2-thioxo-1,2-dihydropyridine-3,5-dicarbonitrile (1.42 g, 4.05 mmol), potassium hydroxide solution (10%) (twice 2.2 mL, 4.05 mmol) and 2-bromo-3'-chloroacetophenone (**9**, 946 mg, 4.05 mmol); reaction time 4.0 h. After dissolving the mixture in acetone (8 mL), silica gel (3 g) was added and the slurry was evaporated to dryness. Column chromatography (ethyl acetate/triethylamine 1:0.05) was performed twice to yield a yellow powder (108 mg, 5%).

Mp: 190–192 °C; IR (KBr): 3465 cm^{-1}, 3347 cm^{-1} and 3207 cm^{-1} (NH), 2217 cm^{-1} (C≡N), 1607 cm^{-1} (C=O); ^{1}H-NMR (DMSO-d_6, 600.1 MHz): δ (ppm) = 2.22 (s, 6H, 2 CH_3), 2.45 (t, J = 7.0 Hz, 2H, CH_2), 3.02 (s, 3H, CH_3), 3.52 (t, J = 7.0 Hz, 2H, CH_2), 6.60 (br s, 2H, NH_2), 6.87 (dd, J = 8.8/2.6 Hz, 1H, ArH), 6.93 (d, J = 2.5 Hz, 1H, ArH), 7.33 (d, J = 8.7 Hz, 1H, ArH), 7.54–7.57 (m, 1H, ArH), 7.60–7.64 (m, 1H, ArH), 7.65–7.68 (m, 2H, ArH), 7.69 (br s, 2H, NH_2); ^{13}C-NMR (DMSO-d_6, 150.9 MHz): δ (ppm) = 39.5 (CH_3), 46.9 (2 CH_3); 50.9, 57.0 (CH_2); 112.2, 112.9, 126.9, 128.1, 131.5, 131.8, 131.9 (CH); 91.9, 99.3, 113.2, 115.2, 116.8, 132.0, 133.3, 142.8, 151.1, 151.6, 151.8, 159.5, 166.3, 185.6 (C); $C_{26}H_{24}Cl_2N_6OS$ (539.48); calcd C 57.89, H 4.48, N 15.58; found C 57.94, H 4.25, N 15.23; MS (EI): m/z (%) = 538.1 $[M]^{+\bullet}$ (74), 139.0 $[M^{+\bullet}$-$C_7H_4ClO]$ (100); isocrat. HPLC: 99.2% at 254 nm and 99.5% at 280 nm, t_{ms} = 6.49 min, t_m = 1.12 min (ACN/buffer pH 2.7, 60:40); λ_{max} (nm): 327, 264.

<u>3,6-Diamino-4-{4-[(2-aminoethyl)(methyl)amino]-2-chlorophenyl}-2-(3-chlorobenzoyl)-thieno[2,3-*b*]pyridine-5-carbonitrile hydrochloride (**4i**)</u>

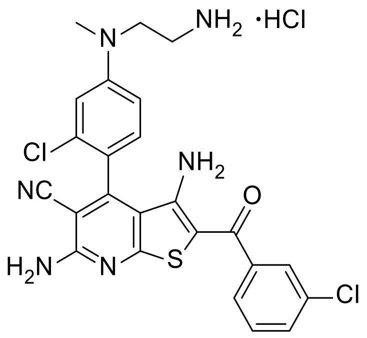

Prepared following GP 5 from *tert*-butyl [2-({3-chloro-4-[3,6-diamino-2-(3-chlorobenzoyl)-5-cyanothieno[2,3-*b*]pyridin-4-yl]phenyl}{methyl}amino)ethyl]carbamate (331 mg, 0.541 mmol), dry dichloromethane (12 mL) and trifluoroacetic acid (7.3 mL); reaction time 14 h; yielding a yellow powder (165 mg, 59%).

Mp: 258–260 °C (dec.); IR (KBr): 3472 cm^{-1}, 3346 cm^{-1} and 3313 cm^{-1} (NH), 2225 cm^{-1} (C≡N), 1623 cm^{-1} (C=O); ^{1}H-NMR (DMSO-d_6, 600.1 MHz): δ (ppm) = 3.05 (s, 3H, CH_3), 3.09 (t, J = 6.9 Hz, 2H, CH_2), 3.64 (t, J = 7.0 Hz, 2H, CH_2), 6.96 (dd, J = 8.8/2.6 Hz, 1H, ArH), 7.07 (d, J = 2.5 Hz, 1H, ArH), 7.40 (d, J = 8.7 Hz, 1H, ArH), 7.53–7.57 (m, 1H, ArH), 7.61–7.67 (m, 3H, ArH), 7.72 (br s, 2H, NH_2, signal disappers after D_2O exchange), 7.84 (br s, 3H, NH_3Cl, signal disappers after D_2O exchange), a further NH_2-signal was not detected; ^{13}C-NMR (DMSO-d_6, 150.9 MHz): δ (ppm) = 38.2 (CH_3); 36.0, 49.0 (CH_2); 111.4, 112.2, 125.6, 126.9, 130.3, 130.6, 130.7 (CH); 91.8, 99.4, 113.2, 115.2, 117.9, 132.1, 133.4, 142.7, 151.0, 151.6, 151.7, 159.5, 166.3, 185.8 (C); $C_{24}H_{21}Cl_3N_6OS$ (547.88); MS (EI): m/z (%) = 510.0 [$M^{+\bullet}$-Cl] (33), 480.0 [$M^{+\bullet}$-CH_5ClN] (100); HRMS (EI): m/z $[M]^{+\bullet}$ calcd 510.07968, found 510.07909; isocrat. HPLC: 99.2% at 254 nm and 99.5% at 280 nm, t_{ms} = 3.75 min, t_m = 1.12 min (ACN/buffer pH 2.7, 20:80); λ_{max} (nm): 328, 262.

3,6-Diamino-2-(3-chlorobenzoyl)-4-[2-chloro-4-(piperazin-1-yl)phenyl]thieno[2,3-*b*]-pyridine-5-carbonitrile hydrochloride (**4j**)

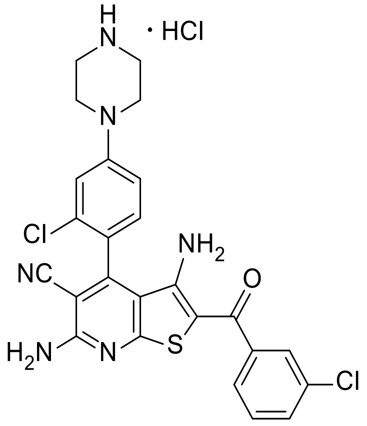

Prepared following GP 5 from *tert*-butyl 4-{3-chloro-4-[3,6-diamino-2-(3-chlorobenzoyl)-5-cyanothieno[2,3-*b*]pyridin-4-yl]phenyl}piperazine-1-carboxylate (102 mg, 0.164 mmol), dry dichlormethane (7 mL) and trifluoroacetic acid (3.3 mL); reaction time 17 h to yield a yellow powder (87 mg, 95%).

Mp: 200–207 °C (dec.); IR (KBr): 3466 cm^{-1} and 3203 cm^{-1} (NH), 2216 cm^{-1} (C≡N), 1603 cm^{-1} (C=O); ^{1}H-NMR (DMSO-d_6, 600.1 MHz): δ (ppm) 3.28–3.32 (m, 4H, 2 CH_2), 3.64–3.72 (m, 4H, 2 CH_2), 6.92 (br s, 2H, NH_2), 7.26 (dd, *J* = 8.7/2.5 Hz, 1H, ArH), 7.41 (d, *J* = 2.5 Hz, 1H, ArH), 7.52 (d, *J* = 8.6 Hz, 1H, ArH), 7.60–7.62 (m, 1H, ArH), 7.67–7.70 (m, 1H, ArH), 7.70–7.74 (m, 2H, ArH), 7.80 (br s, 2H, NH_2), 9.44 (s, 2H, NH_2^+); ^{13}C-NMR (DMSO-d_6, 150.9 MHz): δ (ppm) = 43.1 (2 CH_2), 45.0 (2 CH_2), 114.0, 115.1, 125.4, 126.6, 130.1, 130.3, 130.5 (CH); 91.7, 99.4, 113.0, 115.1, 120.6, 132.1, 133.4, 142.7, 151.3, 151.5, 152.3, 159.4, 166.3, 185.7 (C); $C_{25}H_{21}Cl_3N_6OS$ (558.06); MS (EI): m/z (%) = 522.1 [$M^{+\bullet}$–Cl] (82), 480.0 [$M^{+\bullet}$–78.1]$^{+\bullet}$ (100); HRMS (EI): m/z [M]$^{+\bullet}$ calcd 523.0870, found 523.0869; isocrat. HPLC: 96.4% at 254 nm and 98.0% at 280 nm, t_{ms} = 6.52 min, t_m = 1.2 min (ACN/buffer pH 2.7, 35:65); λ_{max} (nm): 328, 257.

4. Syntheses of Substituted Benzaldehydes

2-Chloro-4-ethoxybenzaldehyde

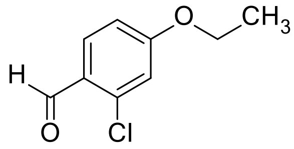

Prepared following GP 1 from 2-chloro-4-hydroxybenzaldehyde (613 mg, 3.92 mmol), K_2CO_3 (1.08 g, 7.84 mmol) and iodoethane (611 mg, 3.92 mmol) in acetonitrile (11 mL); reaction time 18 h. Purification by column chromatography (toluoene/ethyl acetate 2:1) yielded a brown oil (480 mg, 52%).

$C_9H_9ClO_2$ (184.62); ^{1}H-NMR (DMSO-d_6, 400.4 MHz): δ (ppm) = 1.45 (t, 3H, J = 7.0 Hz, CH_3), 4.11 (q, 2H, J = 7.0 Hz, CH_2), 6.88 (dd, 1H, J = 8.8/2.4 Hz, ArH), 6.92 (d, 1H, J = 2.4 Hz, ArH), 7.88 (d, 1H, J = 8.8 Hz, ArH), 10.3 (br s, 1H, CHO). The spectroscopic data coincide with the data given in the literature*.

* Luehr, S.; Vilches-Herrera, M.; Fierro, A.; Ramsay, R. R.; Edmondson, D. E.; Reyes-Parada, M.; Cassels, B. K.; Iturriaga-Vasquez, P., *Bioorg. Med. Chem.* **2010**, *18*, 1388-1395).

4-(Benzyloxy)-2-chlorobenzaldehyde

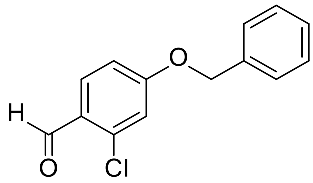

Prepared following GP 1 from 2-chloro-4-hydroxybenzaldehyde (790 mg, 5.04 mmol), K_2CO_3 (1.39 g, 10.1 mmol) and benzyl bromide (900 mg, 5.26 mmol) in acetonitrile (15 mL); reaction time: 16 h. Purification by column chromatography (toluene/ethyl acetate 2:1) yielded a brown powder (925 mg, 75%).

Mp: 65–67 °C (Lit.*: 68–69 °); $C_{14}H_{11}ClO_2$ (246.69); ^{1}H-NMR (DMSO-d_6, 400.4 MHz): δ (ppm) = 5.26 (s, 2H, CH_2), 7.17 (dd, 1H, J = 8.7/2.3 Hz, ArH), 7.29 (d, 1H, J = 2.4 Hz, ArH), 7.33–7.39 (m, 1H, ArH), 7.39–7.45 (m, 2H, ArH), 7.45–7.51 (m, 2H, ArH), 7.84 (d, 1H, J = 8.7 Hz, ArH), 10.2 (s, 1H, CHO); ^{13}C-NMR (DMSO-d_6,

100.7 MHz): δ (ppm) = 70.1 (CH_2); 114.9, 116.1, 127.9 (2 C), 128.1, 128.5 (2 C), 131.3, 188.2 (CH); 125.5, 135.8, 138.2, 163.5 (C).

* Kimachi, T.; Kawase, M.; Matsuki, S.; Tanaka, K.; Yoneda, F., *J. Chem. Soc., Perkin Trans. 1* **1990**, 253-256.

2-Chloro-4-(2-hydroxyethoxy)benzaldehyde

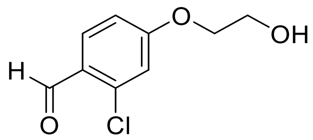

Prepared following GP 1 from 2-chloro-4-hydroxybenzaldehyde (783 mg, 5.00 mmol), K_2CO_3 (1.38 g, 10.0 mmol) and 2-chloroethanol (1.5 mL, 7.5 mmol) in 1,4-dioxane (7.5 mL); reaction time 6 h. Purification by column chromatography (petrol ether/ethyl acetate 3:1) yielded a white powder (725 mg, 72%).

Mp: 82–86 °C; IR (KBr): 3449 cm^{-1} (OH), 1679 cm^{-1} (C=O); ^{1}H-NMR (DMSO-d_6, 600.1 MHz): δ (ppm) = 3.71–3.74 (m, 2H, CH_2), 4.13–4.16 (m, 2H, CH_2), 4.96 (s, 1H, OH), 7.08–7.10 (dd, J = 8.7/2.6 Hz, 1H, ArH), 7.18–7.20 (d, J = 2.4 Hz, 1H, ArH), 7.82–7.84 (d, J = 8.7 Hz, 1H, ArH), 10.2 (s, 1H, CHO); ^{13}C-NMR (DMSO-d_6, 150.9 MHz): δ (ppm) = 59.2, 70.7 (CH_2); 114.6, 115.7, 131.3, 188.1 (CH); 125.3, 138.2, 164.0 (C); $C_9H_9ClO_3$ (200.62); calcd C 53.88, H 4.52; found C 53.98, H 4.50; MS (EI): m/z (%) = 200.0 $[M]^{+\bullet}$ (32), 155.0 $[M^{+\bullet}$-$C_2H_5O]$ (100); isocrat. HPLC: 99.3% at 254 nm and 99.6% at 280 nm, t_{ms} = 3.76 min, t_m = 1.12 min (ACN/H_2O 60:40); λ_{max} (nm): 279, 229, 395; gradient HPLC: 97.6% at 254 nm, t_{ms} = 8.39 min, t_m = 1.22 min (0–2 min: ACN/H_2O 10:90, 2–12 min: ACN/H_2O 10:90 → 90:10, 12–20 min: ACN/H_2O 90:10).

2-Chloro-4-[(2,2-dimethyl-1,3-dioxolan-4-yl)methoxy]benzaldehyde

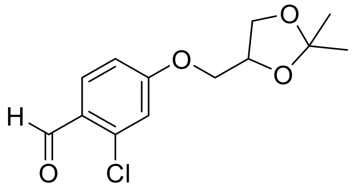

Prepared following GP 1 from 2-chloro-4-hydroxybenzaldehyde (1.25 g, 8.00 mmol), Cs_2CO_3 (7.82 g, 24.0 mmol) and 4-(chloromethyl)-2,2-dimethyl-1,3-dioxolan

(2.27 mL, 16.0 mmol) in DMF (7 mL). Purification by column chromatography (petrol ether/ethyl acetate/triethyl amine 3:1:0.05) yielded a colourless oil (1.22 g, 56%).

IR (KBr): 1685 cm^{-1} (C=O); ^{1}H-NMR (DMSO-*d*$_6$, 600.1 MHz): δ (ppm) = 1.31 (s, 3H, CH_3), 1.36 (s, 3H, CH_3), 3.75–3.79 (m, 1H, CH_2), 4.09–4.13 (m, 2H, CH_2), 4.20–4.22 (m, 1H, CH_2), 4.41–4.45 (m, 1H, CH), 7.11 (dd, *J* = 8.7/2.4 Hz, 1H, ArH), 7.23 (d, *J* = 2.4 Hz, 1H, ArH), 7.83 (d, *J* = 8.7 Hz, 1H, ArH), 10.2 (s, 1H, CHO); ^{13}C-NMR (DMSO-*d*$_6$, 151.0 MHz): δ (ppm) = 25.1, 26.3 (CH_3); 65.2, 69.4 (CH_2); 73.1, 114.5, 115.6, 131.1, 188.0 (CH); 109.0, 125.5, 138.2, 163.5 (C); $C_{13}H_{15}ClO_4$ (270.71); calcd C 57.68, H 5.59; found C 57.94, H 5.68; MS (EI): m/z (%) = 270.1 $[M]^{+\bullet}$ (28), 255.1 $[M^{+\bullet}$-$CH_3]$ (100); isocrat. HPLC: 98.5% at 254 nm and 97.1% at 280 nm, t_{ms} = 3.7 min, t_m = 1.12 min (ACN/H_2O 60:40); λ_{max} (nm): 322, 269, 291; gradient HPLC: 99.8% at 254 nm, t_{ms} = 11.6 min, t_m = 1.25 min (0–2 min: ACN/H_2O 10:90, 2–12 min: ACN/H_2O 10:90 → 90:10, 12–20 min: ACN/H_2O 90:10).

tert-Butyl 2-(3-chloro-4-formylphenoxy)acetate

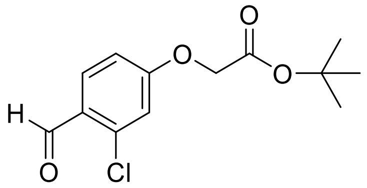

Prepared following GP 1 from 2-chloro-4-hydroxybenzaldehyde (627 mg, 4.00 mmol), Cs_2CO_3 (2.62 g, 8.04 mmol) and *tert*-butyl 2-bromoacetate (714 µL, 4.84 mmol) in DMF (5 mL). Purification by column chromatography (petrol ether/ethyl acetate/triethylamine 2:1:0.05) yielded a white powder (936 mg, 86%).

Mp: 86–88 °C; IR (KBr): 1744 cm^{-1} (C=O); ^{1}H-NMR (DMSO-*d*$_6$, 600.1 MHz): δ (ppm) = 1.43 (s, 9H, 3 CH_3), 4.87 (s, 2H, CH_2), 7.08 (dd, *J* = 8.7/2.5 Hz, 1H, ArH), 7.18 (d, *J* = 2.5 Hz, 1H, ArH), 7.84 (d, *J* = 8.7 Hz, 1H, ArH), 10.2 (s, 1H, CHO); ^{13}C-NMR (DMSO-*d*$_6$, 151.0 MHz): δ (ppm) = 27.6 (3 CH_3); 65.3 (CH_2); 114.6, 116.0, 131.3, 188.2 (CH); 81.9, 125.8, 138.0, 162.7, 167.0 (C); $C_{13}H_{15}ClO_4$ (270.71); calcd C 57.68, H 5.59; found C 57.69, H 5.53; MS (EI): m/z (%) = 270.1 $[M]^{+\bullet}$ (23), 57.1 $[M^{+\bullet}$-$C_4H_9]$ (100); isocrat. HPLC: 98.8% at 254 nm and 99.6% at 280 nm, t_{ms} = 4.2 min, t_m = 1.12 min (ACN/H_2O 60:40); λ_{max} (nm): 273, 261, 230; gradient HPLC: 98.1% at 254 nm, t_{ms} = 12.4 min, t_m = 1.25 min (0–2 min: ACN/H_2O 10:90, 2–12 min: ACN/H_2O 10:90 → 90:10, 12–20 min: ACN/H_2O 90:10).

2-Chloro-4-(dimethylamino)benzaldehyde

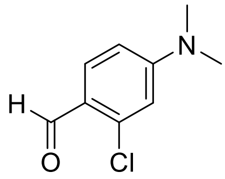

Prepared following GP 2 from 2-chloro-4-fluorobenzaldehyde (805 mg, 5.10 mmol), K_2CO_3 (1.12 g, 8.00 mmol) and dimethylamine hydrochloride (615 mg, 7.51 mmol). Crystallization from water yielded a white powder (779 mg, 85%).

Mp: 78–79 °C (Lit.*: 77–80 °C); IR (KBr): 1663 cm^{-1} (C=O); ^{1}H-NMR (DMSO-d_6, 400.1 MHz): δ (ppm) = 3.06 (s, 6H, 2 CH_3), 6.73 (d, *J* = 2.5 Hz, 1H, ArH), 6.76 (dd, *J* = 8.9/2.5 Hz, 1H, ArH), 7.68 (d, *J* = 8.9 Hz, 1H, ArH), 10.0 (s, 1H, CHO); ^{13}C-NMR (DMSO-d_6, 100.9 MHz): δ (ppm) = 39.5 (2 CH_3); 110.3, 111.0, 130.5, 186.7 (CH); 119.8, 138.7, 154.5 (C); $C_9H_{10}ClNO$ (183.64); calcd C 58.87, H 5.49, N 7.63; found C 58.90, H 5.44, N 7.65.

* Yongpruksa, N.; Pandey, S.; Baker, G. A.; Harmata, M., *Org. Biomol. Chem.* **2011**, *9*, 7979-7982.

2-Chloro-4-(diethylamino)benzaldehyde

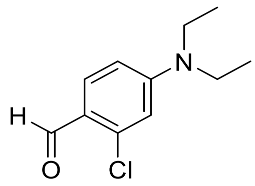

Prepared following GP 2 from 2-chloro-4-fluorobenzaldehyde (1.57 g, 10.0 mmol), K_2CO_3 (2.2 g, 16.1 mmol) and diethylamine (1.57 mL, 15.1 mmol). Crystallization from water yielded a yellow powder (757 mg, 36%).

Mp: 27–28 °C (Lit.*: 25 °C); IR (KBr): 1665 cm^{-1} (C=O); ^{1}H-NMR (DMSO-d_6, 400.1 MHz): δ (ppm) = 1.12 (t, *J* = 7.0 Hz, 6H, 2 CH_3), 3.44 (q, *J* = 7.0 Hz, 4H, 2 CH_2), 6.68–6.70 (d, *J* = 2.5 Hz, 1H, ArH), 6.73–6.75 (dd, *J* = 9.0/2.7 Hz, 1H, ArH), 7.65–7.67 (d, *J* = 8.9 Hz, 1H, ArH), 10.0 (s, 1H, CHO); ^{13}C-NMR (DMSO-d_6, 100.9 MHz): δ (ppm) = 12.1 (2 CH_3); 44.0 (2 CH_2); 110.1, 110.5, 130.9, 186.4 (CH); 119.4, 139.1, 152.3 (C); $C_{11}H_{14}ClNO$ (211.08); MS (EI): m/z (%) = 211.1 $[M]^{+\bullet}$ (29), 196.1 $[M^{+\bullet}-CH_3]$ (100); isocrat. HPLC: 98.6% at 254 nm and 94.9% at 280 nm, t_{ms} =

7.69 min, t_m = 1.12 min (ACN/H_2O 60:40); λ_{max} (nm): 370, 347, 331; gradient HPLC: 97.1% at 254 nm, t_{ms} = 12.5 min, t_m = 1.22 min (0–2 min: ACN/H_2O 10:90, 2–12 min: ACN/H_2O 10:90 → 90:10, 12–20 min: ACN/H_2O 90:10).

* Yongpruksa, N.; Pandey, S.; Baker, G. A.; Harmata, M., *Org. Biomol. Chem.* **2011**, *9*, 7979-7982.

2-Chloro-4-(pyrrolidin-1-yl)benzaldehyde

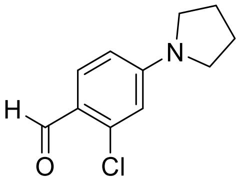

Prepared following GP 2 from 2-chloro-4-fluorobenzaldehyde (792 mg, 5.00 mmol), K_2CO_3 (1.13 g, 8.01 mmol) and pyrrolidine (652 µL, 7.50 mmol). Purification by column chromatography (petrol ether/ethyl acetate 1:1) yielded a yellow powder (875 mg, 83%).

Mp: 90–92 °C (Lit.*: 78–80 °C); IR (KBr): 1655 cm^{-1} (C=O); ^{1}H-NMR (DMSO-d_6, 600.1 MHz): δ (ppm) = 1.89–2.04 (m, 4H, 2 CH_2), 3.30–3.38 (m, 4H, 2 CH_2), 6.58–7.63 (m, 2H, ArH), 7.67 (d, 1H, ArH), 10.0 (s, 1H, CHO); ^{13}C-NMR (DMSO-d_6, 150.9 MHz): δ (ppm) = 24.8 (2 CH_2); 47.5 (2 CH_2); 110.8, 111.2, 130.8, 186.6 (CH); 119.5, 138.7, 151.9 (C); $C_{11}H_{12}ClN_2O_3$ (209.67); calcd C 63.01, H 5.77, N 6.68; found C 62.92, H 5.63, N 6.53.

* Yongpruksa, N.; Pandey, S.; Baker, G. A.; Harmata, M., *Org. Biomol. Chem.* **2011**, *9*, 7979-7982.

2-Chloro-4-(piperidin-1-yl)benzaldehyde

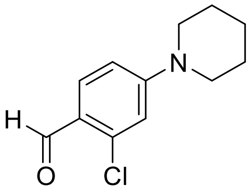

Prepared following GP 2 from 2-chloro-4-fluorobenzaldehyde (805 mg, 5.08 mmol), K_2CO_3 (1.12 g, 8.00 mmol) and piperidine (740 µL, 7.51 mmol). Purification by column chromatography (petrol ether/ethyl acetate 1:1) yielded a yellow oil (535 mg, 48%).

IR (KBr): 1673 cm^{-1} (C=O); ^{1}H-NMR (DMSO-d_6, 600.1 MHz): δ (ppm) = 1.51–1.60 (m, 6H, 3 CH_2), 3.45–3.49 (m, 4H, 2 CH_2), 6.94–6.99 (m, 2H, ArH), 7.67 (d, 1H, ArH), 10.0 (s, 1H, CHO); ^{13}C-NMR (DMSO-d_6, 151.0 MHz): δ (ppm) = 23.6 (CH_2), 24.6 (2 CH_2), 47.2 (2 CH_2); 111.7, 112.6, 130.7, 186.6 (CH); 120.5, 139.2, 154.6 (C). The spectroscopic data coincide with the data given in the literature*.

* Yongpruksa, N.; Pandey, S.; Baker, G. A.; Harmata, M., *Org. Biomol. Chem.* **2011**, *9*, 7979-7982.

<u>2-Chloro-4-morpholinobenzaldehyde</u>

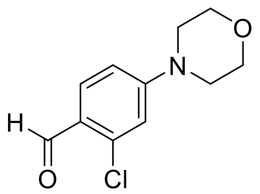

Prepared following GP 2 from 2-chloro-4-fluorobenzaldehyde (1.59 g, 10.0 mmol), K_2CO_3 (2.2 g, 16.0 mmol) and morpholine (1.31 mL, 15.0 mmol). Crystallization from water yielded a yellow powder (1.97 g, 87%).

Mp: 85–89 °C (Lit.*: 87 °C); IR (KBr): 1656 cm^{-1} (C=O); ^{1}H-NMR (DMSO-d_6, 600.1 MHz): δ (ppm) = 3.37–3.42 (m, 4H, 2 CH_2), 3.69–3.74 (m, 4H, 2 CH_2), 6.99–7.05 (m, 2H, ArH), 7.68–7.75 (m, 1H, ArH), 10.0 (s, 1H, CHO); ^{13}C-NMR (DMSO-d_6, 150.9 MHz): δ (ppm) = 46.3 (2 CH_2), 65.7 (2 CH_2); 112.1, 113.3, 130.7, 187.2 (CH); 121.7, 138.7, 155.1 (C); $C_{11}H_{12}ClNO_2$ (225.67); calcd C 58.55, H 5.36, N 6.21; found C 58.40, H 5.37, N 6.05; isocrat. HPLC: 99.1% at 254 nm and 98.1% at 280 nm, t_{ms} = 3.23 min, t_m = 1.12 min (ACN/H_2O 50:50); λ_{max} (nm): 353, 347, 328; gradient HPLC: 97.3% at 254 nm, t_{ms} = 10.3 min, t_m = 1.22 min (0–2 min: ACN/H_2O 10:90, 2–12 min: ACN/H_2O 10:90 → 90:10, 12–20 min: ACN/H_2O 90:10).

* Yongpruksa, N.; Pandey, S.; Baker, G. A.; Harmata, M., *Org. Biomol. Chem.* **2011**, *9*, 7979-7982.

<u>*tert*-Butyl {2-[(3-chloro-4-formylphenyl)(methyl)amino]ethyl}carbamate</u>

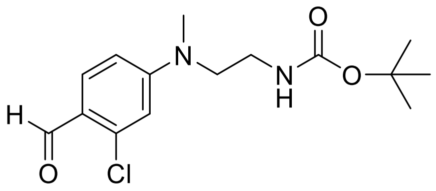

Prepared following GP 2 from 2-chloro-4-fluorobenzaldehyde (480 mg, 3.03 mmol), K_2CO_3 (663 mg, 4.80 mmol) and *tert*-butyl 2-(methylamino)ethylcarbamate hydrochloride (995 mg, 4.70 mmol). Purification by column chromatography (toluene/ethyl acetate 2:1) yielded a white powder (780 mg, 85%).

Mp: 25–27 °C; IR (KBr): 1673 cm^{-1} (C=O), 3367 cm^{-1} (NH); ^{1}H-NMR (DMSO-d_6, 600.1 MHz): δ (ppm) = 1.32 (s, 9H, 3 CH_3), 3.01 (s, 3H, CH_3), 3.12 (t, *J* = 6.2 Hz, 2H, CH_2), 3.49 (t, *J* = 6.3 Hz, 2H, CH_2), 6.77 (dd, *J* = 8.9/6.0 Hz, 1H, ArH), 6.93 (d, *J* = 6.0 Hz, 1H, ArH), 7.64 (d, *J* = 8.9 Hz, 1H, ArH), 10.0 (s, 1H, CHO); ^{13}C-NMR (DMSO-d_6, 150.9 MHz): δ (ppm) = 28.1 (3 CH_3), 38.1 (CH_3); 37.3, 50.7 (CH_2); 110.3, 111.0, 130.6, 186.7 (CH); 77.2, 119.8, 138.8, 154.1, 155.7 (C); $C_{15}H_{21}ClN_2O_3$ (312.79); calcd C 57.60, H 6.77, N 8.96; found C 58.0, H 6.71, N 8.60; MS (EI): m/z (%) = 312.2 $[M]^{+\bullet}$ (10), 182.1 $[M^{+\bullet}$-$C_6H_{12}NO_2]$ (100); isocrat. HPLC: 96.8% at 254 nm and 98.2% at 280 nm, t_{ms} = 6.01 min, t_m = 1.12 min (ACN/H_2O 45:55); λ_{max} (nm): 326, 271, 293; gradient HPLC: 97.8% at 254 nm, t_{ms} = 11.6 min, t_m = 1.25 min (0–2 min: ACN/H_2O 10:90, 2–12 min: ACN/H_2O 10:90 → 90:10, 12–20 min: ACN/H_2O 90:10).

<u>*tert*-Butyl 4-(3-chloro-4-formylphenyl)piperazine-1-carboxylate</u>

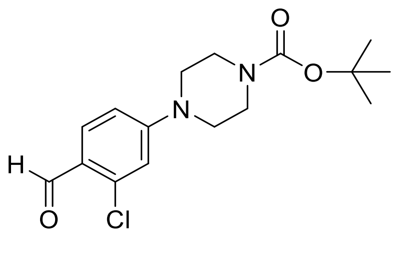

Prepared following GP 2 from 2-chloro-4-fluorobenzaldehyde (803 mg, 5.06 mmol), K_2CO_3 (1.13 g, 8.01 mmol) and *tert*-Butyl piperazine-1-carboxylate (1.40 g, 7.50 mmol). Purification by column chromatography (toluene/ethyl acetate 2:1) yielded a white powder (1.62 g, 91%).

Mp: 95–102 °C; IR (KBr): 1689 cm^{-1} (C=O); ^{1}H-NMR (DMSO-d_6, 600.1 MHz): δ (ppm) = 1.43 (s, 9H, 3 CH_3), 3.42–3.48 (m, 8H, 4 CH_2), 6.96–7.02 (m, 2H, ArH), 7.67–7.73

(m, 1H, ArH), 10.1 (s, 1H, CHO); ^{13}C-NMR (DMSO-d_6, 150.9 MHz): δ (ppm) = 28.0 (3 CH_3); 45.8 (4 CH_2); 112.2, 113.3, 130.9, 187.2 (CH); 79.2, 121.4, 138.8, 153.8, 154.6 (C); $C_{16}H_{21}ClN_2O_3$ (324.81); calcd C 59.17, H 6.52, N 8.62; found C 59.10, H 6.39, N 8.30; MS (EI): m/z (%) = 324.1 $[M]^{+\bullet}$ (8), 182.0 $[M^{+\bullet}$-142.8] (100); isocrat. HPLC: 98.3% at 254 nm and 98.3% at 280 nm, t_{ms} = 4.81 min, t_m = 1.12 min (ACN/H_2O 60:40); λ_{max} (nm): 353, 347, 333; gradient HPLC: 98.3% at 254 nm, t_{ms} = 12.7 min, t_m = 1.22 min (0–2 min: ACN/H_2O 10:90, 2–12 min: ACN/H_2O 10:90 → 90:10, 12–20 min: ACN/H_2O 90:10).

<u>2-Chloro-4-{[2-(dimethylamino)ethyl][methyl]amino}benzaldehyde</u>

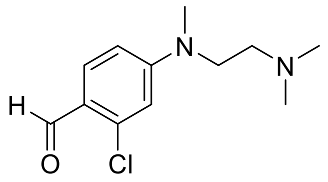

Prepared following GP 2 from 2-chloro-4-fluorobenzaldehyde (794 mg, 5.01 mmol), K_2CO_3 (1.12 g, 8.00 mmol) and *N*[1],*N*[1],*N*[2]-Trimethylethan-1,2-diamine (773 µL, 7.51 mmol). Purification by column chromatography (toluene/ethyl acetate/triethylamine 3:1:0.05) yielded a brown oil (429 mg, 36%).

IR (KBr): 1667 cm^{-1} (C=O); ^{1}H-NMR (DMSO-d_6, 600.1 MHz): δ (ppm) = 2.18 (s, 6H, 2 CH_3), 2.39 (t, 2H, J = 6.9 Hz, CH_2), 3.04 (s, 3H, CH_3), 3.54 (t, 2H, J = 6.9 Hz, CH_2), 6.71 (d, 1H, J = 2.5 Hz, ArH), 6.77 (dd, 1H, J = 9.0/2.5 Hz, ArH), 7.67 (d, 1H, J = 8.9 Hz, ArH), 10.0 (s, 1H, CHO); ^{13}C-NMR (DMSO-d_6, 150.9 MHz): δ (ppm) = 38.5 (CH_3), 45.5 (2 CH_3); 49.5, 55.8 (CH_2); 111.4, 112.0, 131.8, 187.7 (CH); 119.7, 138.9, 153.7 (C); $C_{12}H_{17}ClN_2O$ (240.73); calcd C 59.87, H 7.12, N 11.64; found C 59.60, H 7.39, N 11.36; MS (EI): m/z (%) = 240.1 $[M]^{+\bullet}$ (26), 182.1 $[M^{+\bullet}$-$C_3H_8N]$ (67), 58.1 $[M^{+\bullet}$-$C_9H_9ClNO]$ (100); isocrat. HPLC: 98.5% at 254 nm and 97.1% at 280 nm, t_{ms} = 5.48 min, t_m = 1.12 min (ACN/buffer pH 2.7, 20:80); λ_{max} (nm): 354, 348, 255.

5. Syntheses of 6-Amino-4-aryl-2-thioxo-1,2-dihydropyridine-3,5-dicarbonitriles

6-Amino-4-(2,4-dichlorophenyl)-2-thioxo-1,2-dihydropyridine-3,5-dicarbonitrile

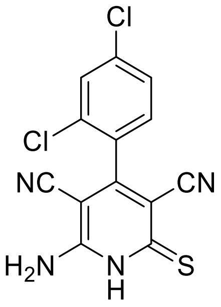

Prepared following GP 3 from 2,4-dichlorobenzaldehyde (705 mg, 4.02 mmol), 2-cyanothioacetamide (**7**, 400 mg, 3.99 mmol) and malonodinitrile (**6**, 278 mg, 4.21 mmol). Workup by column chromatogaphy (petrol ether/ethyl acetate/acetic acid 2:1:0.05) yielded a yellow powder (333 mg, 26%) which was used for subsequent synthetic procedures without further purification.

Mp: 240–244 °C (Lit.*: 257–259 °C); $C_{13}H_6Cl_2N_4S$ (321.18); IR (KBr): 3386 cm^{-1}, 3311 cm^{-1} and 3206 cm^{-1}(NH), 2220 cm^{-1} (C≡N), ^{1}H-NMR (DMSO-*d*$_6$, 600.1 MHz): δ (ppm) = 7.59 (d, *J* = 8.3 Hz, 1H, ArH), 7.67 (dd, *J* = 8.3/2.0 Hz, 1H, ArH), 7.92 (d, *J* = 2.0 Hz, 1H, ArH), 8.30 (br s, 2H, NH_2), 13.23 (br s, 1H, NH); ^{13}C-NMR (DMSO-*d*$_6$, 150.9 MHz): δ (ppm) = 128.2, 129.4, 131.0 (CH); 81.6, 102.9, 113.8, 115.6, 131.7, 132.4, 135.7, 154.3, 155.3, 179.3 (C).

* Zhuang, Q.-Y.; Xu, J.-N.; Tu, S.-J.; Jia, R.-H.; Zhang, J.-Y.; Li, C.-M.; Zhou, D.-X.,*Chin. J. Chem.* **2007**, *25*, 1568-1572.

6-Amino-4-(2-chloro-4-fluorophenyl)-2-thioxo-1,2-dihydropyridine-3,5-dicarbonitrile

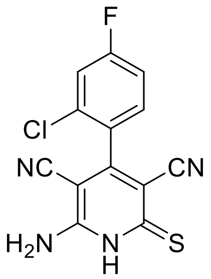

Prepared following GP 3 from 2-chloro-4-fluorobenzaldehyde (476 mg, 3.00 mmol), 2-cyanothioacetamide (**7**, 302 mg, 3.02 mmol) and malonodinitrile (**6**, 207 mg, 3.13 mmol). Workup by column chromatogaphy (petrol ether/ethyl acetate/acetic acid

2:1:0.05) yielded a yellow powder (644 mg, 56%) which was used for subsequent synthetic procedures without further purification.

Mp: 241–243 °C; $C_{13}H_6ClFN_4S$ (304.72); IR (KBr): 3385 cm^{-1}, 3313 cm^{-1} and 3203 cm^{-1}(NH), 2220 cm^{-1} (C≡N), ^{1}H-NMR (DMSO-d_6, 600.1 MHz): δ (ppm) = 7.47 (dd, J = 8.5/2.6 Hz, 1H, ArH), 7.63 (d, J = 8.6 Hz, 1H, ArH), 7.75 (d, J = 2.5 Hz, 1H, ArH), 8.29 (br s, 2H, NH_2), 13.21 (br s, 1H, NH); ^{13}C-NMR (DMSO-d_6, 150.9 MHz): δ (ppm) = 115.3 (d, $^2J_{C,F}$ = 21.8 Hz), 117.3 (d, $^2J_{C,F}$ = 25.7 Hz), 131.5 (d, $^3J_{C,F}$ = 9.4 Hz) (CH); 81.9, 103.2, 113.8, 115.6, 129.9 (d, $^4J_{C,F}$ = 3.4 Hz), 131.9 (d, $^3J_{C,F}$ = 11.1 Hz), 154.2, 155.5, 162.6 (d, $^1J_{C,F}$ = 251 Hz), 179.3 (C).

6-Amino-4-(4-bromo-2-chlorophenyl)-2-thioxo-1,2-dihydropyridine-3,5-dicarbonitrile

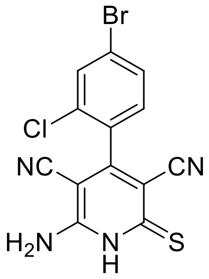

Prepared following GP 3 from 2-chloro-4-bromobenzaldehyde (441 mg, 2.01 mmol), 2-cyanothioacetamide (**7**, 206 mg, 2.06 mmol) and malonodinitrile (**6**, 141 mg, 2.13 mmol). Workup by column chromatogaphy (petrol ether/ethyl acetate/acetic acid 2:1:0.05) yielded a yellow powder (450 mg, 61%) which was used for subsequent synthetic procedures without further purification.

6-Amino-4-(2-chloro-4-methylphenyl)-2-thioxo-1,2-dihydropyridine-3,5-dicarbonitrile

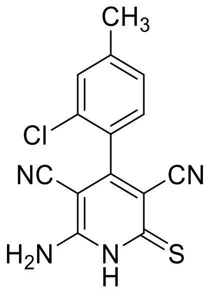

Prepared following GP 3 from 2-chloro-4-methylbenzaldehyde (311 mg, 2.01 mmol), 2-cyanothioacetamide (**7**, 202 mg, 2.02 mmol) and malonodinitrile (**6**, 138 mg, 2.03 mmol). Workup by column chromatogaphy (petrol ether/ethyl acetate/acetic acid

2:1:0.05) yielded a yellow powder (306 mg, 51%) which was used for subsequent synthetic procedures without further purification.

6-Amino-4-(2-chloro-4-methoxyphenyl)-2-thioxo-1,2-dihydropyridine-3,5-dicarbonitrile

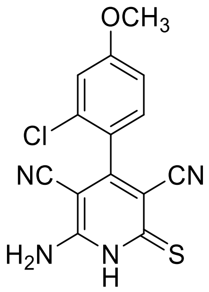

Prepared following GP 3 from 2-chloro-4-methoxybenzaldehyde (343 mg, 2.01 mmol), 2-cyanothioacetamide (**7**, 203 mg, 2.03 mmol) and malonodinitrile (**6**, 144 mg, 2.18 mmol). Workup by column chromatogaphy (petrol ether/ethyl acetate/acetic acid 3:1:0.05) yielded a yellow powder (233 mg, 36.6%) which was used for subsequent synthetic procedures without further purification.

6-Amino-4-(2-chloro-4-ethoxyphenyl)-2-thioxo-1,2-dihydropyridine-3,5-dicarbonitrile

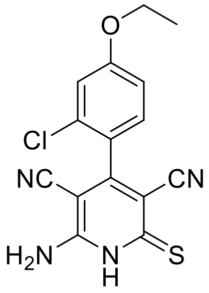

Prepared following GP 3 from 2-chloro-4-ethoxybenzaldehyde (480 mg, 2.61 mmol), 2-cyanothioacetamide (**7**, 267 mg, 2.66 mmol) and malonodinitrile (**6**, 174 mg, 2.63 mmol). Workup by column chromatogaphy (petrol ether/ethyl acetate/acetic acid 5:1:0.05) yielded a yellow powder (177 mg, 21%) which was used for subsequent synthetic procedures without further purification.

Mp: 239–243 °C; $C_{15}H_{11}ClN_4OS$ (330.79); ^{1}H-NMR (DMSO-d_6, 400.4 MHz): δ (ppm) = 1.63 (t, J = 7.0 Hz, 3H, CH_3), 3.13 (q, J = 7.0, 2H, CH_2), 7.08 (dd, J = 8.7/2.5 Hz, 1H, ArH), 7.23 (d, J = 2.4 Hz, 1H, ArH), 7.41 (d, J = 8.6 Hz, 1H, ArH), 13.13 (br s, 1H, NH), the NH_2 group was not detected; ^{13}C-NMR (DMSO-d_6, 100.7 MHz): δ (ppm) = 14.5 (CH_3); 63.9 (CH_2); 114.2, 115.3, 130.6 (CH), 82.4, 103.6, 114.0, 115.9, 125.2, 131.5, 154.3, 156.5, 160.4, 179.3 (C).

6-Amino-4-[4-(benzyloxy)-2-chlorophenyl]-2-thioxo-1,2-dihydropyridine-3,5-dicarbonitrile

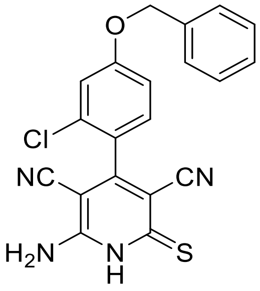

Prepared following GP 3 from 4-(benzyloxy)-2-chlorobenzaldehyde (925 mg, 3.74 mmol), 2-cyanothioacetamide (**7**, 380 mg, 3.79 mmol) and malonodinitrile (**6**, 250 mg, 3.78 mmol). Workup by column chromatogaphy (petrol ether/ethyl acetate/acetic acid 5:1:0.05) yielded a yellow powder (130 mg, 8.8%) which was used for subsequent synthetic procedures without further purification.

Mp: 239–242 °C; $C_{20}H_{13}ClN_4OS$ (392.86); ^{1}H-NMR (DMSO-d_6, 400.4 MHz): δ (ppm) = 5.20 (s, 2H, CH_2), 7.18 (dd, J = 8.6/2.5 Hz, 1H, ArH), 7.36 (d, J = 2.5 Hz, 1H, ArH), 7.37–7.39 (m, 1H, ArH), 7.40–7.47 (m, 3H, ArH), 7.49–7.50 (m, 1H, ArH), 7.50–7.51 (m, 1H, ArH), 13.14 (br s, 1H, NH), the NH_2 group was not detected; ^{13}C-NMR (DMSO-d_6, 100.7 MHz): δ (ppm) = 69.9 (CH_2); 114.4, 115.6, 128.0 (2 C), 128.1, 128.5 (2 C), 130.6 (CH); 82.3, 103.5, 114.0, 115.8, 125.5, 131.4, 136.1, 154.2, 156.4, 160.1, 179.2 (C).

6-Amino-4-[2-chloro-4-(2-hydroxyethoxy)phenyl]-2-thioxo-1,2-dihydropyridine-3,5-dicarbonitrile

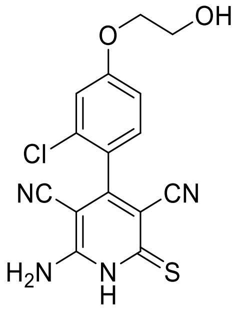

Prepared following GP 3 from 2-chloro-4-(2-hydroxyethoxy)benzaldehyde (327 mg, 1.63 mmol), 2-cyanothioacetamide (**7**, 164 mg, 1.64 mmol) and malonodinitrile (**6**,

107 mg, 1.63 mmol). Workup by column chromatogaphy (petrol ether/ethyl acetate/acetic acid 2:1:0.05) yielded a yellow powder (332 mg, 59%) which was used for subsequent synthetic procedures without further purification.

6-Amino-4-{2-chloro-4-[(2,2-dimethyl-1,3-dioxolan-4-yl)methoxy]phenyl}-2-thioxo-1,2-dihydropyridine-3,5-dicarbonitrile

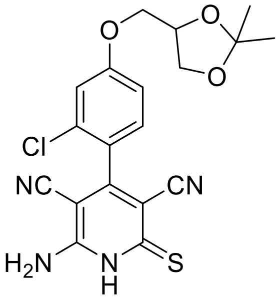

Preparation following GP 3 from 2-chloro-4-[(2,2-dimethyl-1,3-dioxolan-4-yl)methoxy]benzaldehyde (681 mg, 2.52 mmol), 2-cyanothioacetamide (**7**, 252 mg, 2.52 mmol) and malonodinitrile (**6**, 166 mg, 2.51 mmol) yielded 489 mg (59%) of a brown oil which was used for subsequent synthetic procedures without further purification.

tert-Butyl 2-[4-(6-amino-3,5-dicyano-2-thioxo-1,2-dihydropyridin-4-yl)-3-chlorophen-oxy]acetate

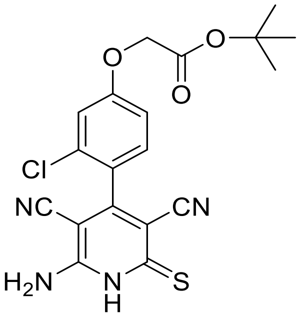

Preparation following GP 3 from *tert*-butyl 2-(3-chloro-4-formylphenoxy)acetate (406 mg, 1.50 mmol), 2-cyanothioacetamide (**7**, 99.1 mg, 1.50 mmol) and malonodinitrile (**6**, 150 mg, 1.50 mmol) yielded a yellow powder (208 mg, 33%) which was used for subsequent synthetic procedures without further purification.

6-Amino-4-[2-chloro-4-(dimethylamino)phenyl]-2-thioxo-1,2-dihydropyridine-3,5-dicarbonitrile

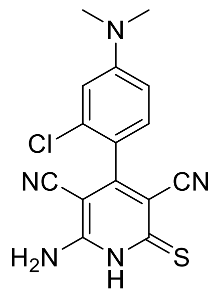

Prepared following GP 3 from 2-chloro-4-(dimethylamino)benzaldehyde (389 mg, 2.12 mmol), 2-cyanothioacetamide (**7,** 212 mg, 2.12 mmol) and malonodinitrile (**6**, 140 mg, 2.12 mmol). Workup by column chromatogaphy (petrol ether/ethyl acetate/acetic acid 2:1:0.05) yielded a yellow oil (372 mg, 53%) which was used for subsequent synthetic procedures without further purification.

6-Amino-4-[2-chloro-4-(diethylamino)phenyl]-2-thioxo-1,2-dihydropyridine-3,5-dicarbonitrile

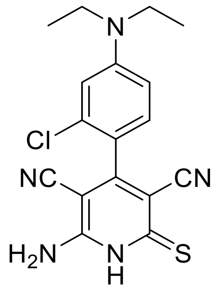

Prepared following GP 3 from 2-chloro-4-(diethylamino)benzaldehyde (425 mg, 2.01 mmol), 2-cyanothioacetamide (**7**, 204 mg, 2.03 mmol) and malonodinitrile (**6**, 144 mg, 2.18 mmol). Workup by column chromatogaphy (petrol ether/ethyl acetate/acetic acid 2:1:0.05) yielded a yellow powder (554 mg, 77%) which was used for subsequent synthetic procedures without further purification.

<u>6-Amino-4-[2-chloro-4-(pyrrolidin-1-yl)phenyl]-2-thioxo-1,2-dihydropyridine-3,5-dicarbonitrile</u>

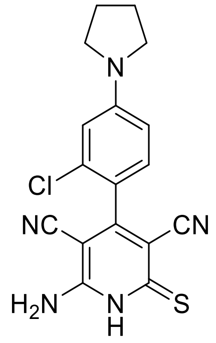

Prepared following GP 3 from 2-chloro-4-(pyrrolidin-1-yl)benzaldehyde (428 mg, 2.04 mmol), 2-cyanothioacetamide (**7**, 200 mg, 2.00 mmol) and malonodinitrile (**6**, 133 mg, 2.01 mmol). Workup by column chromatogaphy (petrol ether/ethyl acetate/acetic acid 2:1:0.05) yielded a yellow powder (536 mg, 73%) which was used for subsequent synthetic procedures without further purification.

<u>6-Amino-4-[2-chloro-4-(piperidin-1-yl)phenyl]-2-thioxo-1,2-dihydropyridine-3,5-dicarbonitrile</u>

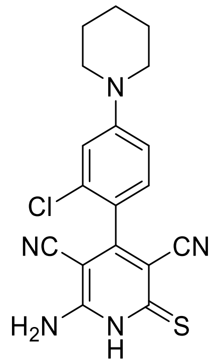

Prepared following GP 3 from 2-chloro-4-(piperidin-1-yl)benzaldehyde (475 mg, 2.12 mmol), 2-cyanothioacetamide (**7**, 212 mg, 2.12 mmol) and malonodinitrile (**6**, 140 mg, 2.12 mmol). Workup by column chromatogaphy (petrol ether/ethyl acetate/acetic acid 2:1:0.05) yielded a yellow powder (372 mg, 47%) which was used for subsequent synthetic procedures without further purification.

6-Amino-4-(2-chloro-4-morpholinophenyl)-2-thioxo-1,2-dihydropyridine-3,5-dicarbonitrile

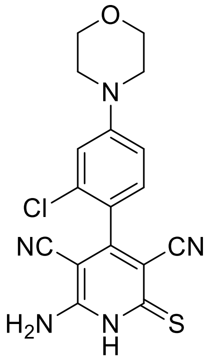

Prepared following GP 3 from 2-chloro-4-morpholinobenzaldehyde (454 mg, 2.01 mmol), 2-cyanothioacetamide (**7**, 205 mg, 2.05 mmol) and malonodinitrile (**6**, 151 mg, 2.29 mmol). Workup by column chromatogaphy (petrol ether/ethyl acetate/acetic acid 2:1:0.05) yielded a yellow powder (512 mg, 68%) which was used for subsequent synthetic procedures without further purification.

tert-Butyl (2-{[4-(6-amino-3,5-dicyano-2-thioxo-1,2-dihydropyridin-4-yl)-3-chloro-phenyl]-[methyl]amino}ethyl)carbamate

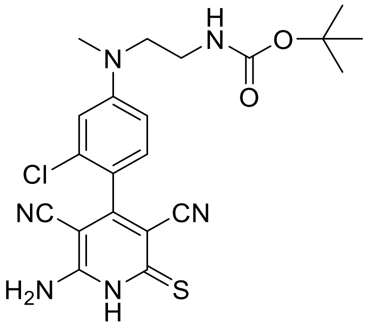

Prepared following GP 3 from *tert*-butyl {2-[(3-chloro-4-formylphenyl)(methyl)amino]ethyl}carbamate (314 mg, 1.00 mmol), 2-cyanothioacetamide (**7**, 101 mg, 1.00 mmol) and malonodinitrile (**6**, 66 mg, 1.01 mmol). Workup by column chromatogaphy (petrol ether/ethyl acetate/acetic acid 3:1:0.05) yielded a yellow powder (228 mg, 50%) which was used for subsequent synthetic procedures without further purification.

tert-Butyl 4-[4-(6-amino-3,5-dicyano-2-thioxo-1,2-dihydropyridin-4-yl)-3-chlorophenyl]-piperazine-1-carboxylate

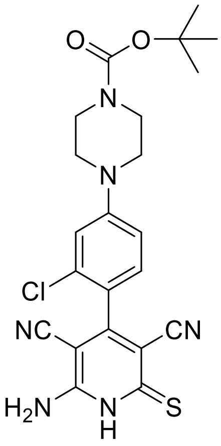

Prepared following GP 3 from *tert*-butyl 4-(3-chloro-4-formylphenyl)piperazine-1-carboxylate (662 mg, 2.04 mmol), 2-cyanothioacetamide (**7**, 210 mg, 2.09 mmol) and malonodinitrile (**6**, 153 mg, 2.32 mmol). Workup by column chromatogaphy (petrol ether/ethyl acetate/acetic acid 3:1:0.05) yielded a brown oil (720 mg, 75%) which was used for subsequent synthetic procedures without further purification.

6-Amino-4-(2-chloro-4-{[2-(dimethylamino)ethyl][methyl]amino}phenyl)-2-thioxo-1,2-dihydropyridine-3,5-dicarbonitrile

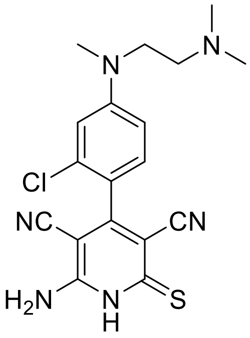

A mixture of 3-(2-chloro-4-{[2-(dimethylamino)ethyl][methyl]amino}phenyl)-2-cyanoprop-2-enthioamide (1.13 g, 3.50 mmol), malonodinitrile (**6**, 232 mg, 3.51 mmol), and piperidine (5 drops) in 1,4-dioxane (3 mL) was stirred for 4 h at 80 °C. The mixture was evaporated to dryness. The residue was used for the subsequent reaction without further purification.

6. Synthesis of a 3-Aryl-2-cyanoprop-2-enthioamide

3-(2-Chloro-4-{[2-(dimethylamino)ethyl][methyl]amino}phenyl)-2-cyanoprop-2-en-thioamide

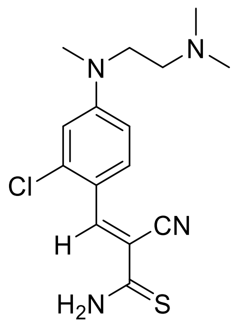

2-Chloro-4-{[2-(dimethylamino)ethyl][methyl]amino}benzaldehyde (600 mg, 2.50 mmol) and 2-cyanothioacetamide (**7**, 252 mg, 2.51 mmol) were dissolved in ethanol (3 mL). One drop of piperidine was added. The mixture was sonicated at room temperature in an ultrasonic bath for 10-15 min. The precipitate was filtered off with suction. Crystallization from ethanol yielded 452 mg (56%) red needles.

Mp: 209–211 °C; IR (KBr): 3347 cm^{-1} (NH), 2198 cm^{-1} (C≡N); ^{1}H-NMR (DMSO-d_6, 600.1 MHz): δ (ppm) = 2.19 (s, 6H, 2 CH_3), 2.42 (t, J = 6.8 Hz, 2H, CH_2), 3.06 (s, 3H, CH_3), 3.56 (t, J = 6.8 Hz, 2H, CH_2), 6.86 (dd, J = 9.1/2.8 Hz, 1H, ArH), 6.88 (d, J = 2.7 Hz, 1H, ArH), 8.20 (d, J = 9.0 Hz, 1H, ArH), 8.48 (s, 1H, CH), 9.29 (br s, 1H, NH_2), 9.91 (br s, 1H, NH_2); ^{13}C-NMR (DMSO-d_6, 150.9 MHz): δ (ppm) = 39.5 (CH_3), 46.5 (2 CH_3); 50.5, 56.9 (CH_2); 111.8, 112.8, 131.2, 146.6 (CH); 105.7, 115.3, 117.3, 138.8, 152.6, 192.5 (C); $C_{15}H_{19}ClN_4S$ (322.86); calcd C 55.80, H 5.93, N 17.35; found C 56.06, H 5.85, N 17.15; MS (EI): m/z (%) = 322.1 $[M]^{+\bullet}$ (6), 58.1 $[C_3H_8N]^{+\bullet}$ (100); isocrat. HPLC: 99.4% at 254 nm and 98.9% at 280 nm, t_{ms} = 4.95 min, t_m = 1.12 min (ACN/buffer pH 2.7, 20:80); λ_{max} (nm): 238, 261.
